# Secondary Envelopment of Human Cytomegalovirus Is a Fast Process Utilizing the Endocytic Compartment as a Major Membrane Source

**DOI:** 10.3390/biom14091149

**Published:** 2024-09-12

**Authors:** Tim Bergner, Laura Cortez Rayas, Gesa Freimann, Clarissa Read, Jens von Einem

**Affiliations:** 1 Central Facility for Electron Microscopy, Ulm University, 89081 Ulm, Germany; tim.bergner@uni-ulm.de (T.B.); gesa.freimann@web.de (G.F.); 2 Institute of Virology, Ulm University Medical Center, 89081 Ulm, Germany; laura.cortez-rayas@uni-ulm.de

**Keywords:** herpesvirus, morphogenesis, plasma membrane labeling, HCMV, TEM, STEM

## Abstract

Secondary envelopment of the human cytomegalovirus (HCMV) is a critical but not well-understood process that takes place at the cytoplasmic viral assembly complex (cVAC) where nucleocapsids acquire their envelope by budding into cellular membranes containing viral glycoproteins. Previous studies presented controversial results regarding the composition of the viral envelope, suggesting trans-Golgi and endosomal origins, as well as intersections with the exosomal and endocytic pathways. Here, we investigated the role of endocytic membranes for the secondary envelopment of HCMV by using wheat germ agglutinin (WGA) pulse labeling to label glycoproteins at the plasma membrane and to follow their trafficking during HCMV infection by light microscopy and transmission electron microscopy (TEM). WGA labeled different membrane compartments within the cVAC, including early endosomes, multivesicular bodies, trans-Golgi, and recycling endosomes. Furthermore, TEM analysis showed that almost 90% of capsids undergoing secondary envelopment and 50% of enveloped capsids were WGA-positive within 90 min. Our data reveal extensive remodeling of the endocytic compartment in the late stage of HCMV infection, where the endocytic compartment provides an optimized environment for virion morphogenesis and serves as the primary membrane source for secondary envelopment. Furthermore, we show that secondary envelopment is a rapid process in which endocytosed membranes are transported from the plasma membrane to the cVAC within minutes to be utilized by capsids for envelopment.

## 1. Introduction

The formation of infectious herpesvirus particles is completed by a cytoplasmic envelopment process in which the capsids acquire a lipid bilayer envelope. This process is referred to as secondary envelopment and is conserved among herpesviruses. Secondary envelopment involves the budding of partially tegumented capsids at intracellular vesicle membranes that contain viral glycoproteins, which drives the engulfment of these capsids by membranes. The envelopment process is completed by membrane fission, resulting in virions enclosed by a vesicle membrane. In addition to infectious virions, HCMV-infected cells produce non-infectious particles called dense bodies (DB), which are enveloped viral protein aggregates without a capsid. Betaherpesviruses, and in particular human cytomegalovirus (HCMV), also known as human herpesvirus 5 (HHV-5), have been shown to induce reorganization of cellular membrane systems, including Golgi, ER, endosomal, and exosomal membranes, to form a cytoplasmic compartment known as the cytoplasmic viral assembly compartment (cVAC), viral assembly compartment (vAC), or assembly compartment (AC) [1,2,3]. The cVAC has a unique structure characterized by a specific arrangement of different membrane compartments. These compartments include Golgi stacks that are reorganized to form an outer ring of membranes defining the region of the cVAC and membranes from the secretory and endosomal compartment that are localized towards the center of the cVAC [1,4,5,6,7,8]. The cVAC forms around the centrosomal microtubule organizing center (MTOC) but also functions as an MTOC for non-centrosomal microtubules [9]. One advantage of forming a compartment such as the cVAC is the concentration of components and parts necessary for the assembly of infectious virions, including capsids, viral proteins, and membranes (reviewed in [10]). The importance of the cVAC for virion assembly is further underscored by the fact that secondary envelopment has only been detected in this region [3] and that defects in cVAC formation result in impaired viral growth [11]. HCMV virions have a complex structure consisting of the DNA genome, an icosahedral capsid, a tegument layer of proteins of viral and cellular origin, and various glycoproteins and glycoprotein complexes embedded in the host cell-derived lipid bilayer envelope [12,13,14]. While the role of tegument proteins and glycoproteins in secondary envelopment is becoming better understood and the underlying molecular mechanisms are gradually being uncovered, it is still a matter of debate from which compartment of the host cell the HCMV envelope originates. Data from fluid phase endocytic tracer horseradish peroxidase (HRP) experiments in combination with electron microscopy indicated budding of HCMV at membranes of tubular early endosomes [15]. In contrast, immunogold labeling of subcellular markers in HCMV-infected cells suggested that secondary envelopment of HCMV particles takes place mainly at Golgi-derived secretory vacuoles [16]. Subsequent work using immunogold labeling on isolated virus particles demonstrated the incorporation of various subcellular markers, such as the trans-Golgi network (TGN) protein TGN46, the endosomal markers early endosomal antigen 1 (EEA1) and annexin I, the recycling endosome marker Transferrin receptor (TfR), the multivesicular body (MVB) marker CD63, and the cation-independent mannose 6-phosphate receptor, into the viral envelope, suggesting the generation of a mixed membrane compartment containing TGN, endosomal, and exosomal markers [17]. Other herpesviruses have also been shown to utilize Golgi membranes for their secondary envelopment [18,19,20,21,22]. Additionally, it has been demonstrated that endocytic tubules serve as the primary membrane source in herpes simplex virus 1 (HSV-1) infections [23].

In addition to the relocation and expansion of membrane compartments, there is evidence of the formation of mixed membrane compartments during HCMV infection, which complicates an unambiguous identification of the HCMV envelope membrane source [4]. There are several lines of evidence that HCMV utilizes membranes of the endocytic compartment for virion assembly. Several HCMV structural proteins, e.g., glycoproteins B, M (gB, gM), and gpUL132 as well as the tegument protein pUL71, are transported to the plasma membrane of the host cell during infection and then, triggered by endocytic trafficking motifs, transported back to the cVAC via endocytosis [24,25,26,27]. For example, the trafficking motifs in the cytoplasmic tail of gM are essential for the formation of infectious virus particles [25]. Similarly, a mutation of the N-terminal YxxΦ trafficking motif of pUL71 results in an altered intracellular localization of this protein at the plasma membrane and a defect in virus assembly [26]. Furthermore, it was shown that endocytosis inhibitors significantly reduce HCMV growth rates without affecting early and late viral gene expression [28]. Dynamin, a key component of the endocytic machinery, has an essential role in the early stages of murine cytomegalovirus (MCMV) infection, involving the rearrangement of membranes to form the cVAC, which is critical for virus maturation [29] and late in infection for virus morphogenesis and egress [30].

Together, these data suggest an important role of endocytosed membranes in the secondary envelopment of HCMV and support the hypothesis that the endocytic compartment is the primary source of HCMV envelopment. To better understand the use and extent of viral utilization of the endocytic membrane compartment for the cytoplasmic envelopment of capsids, we characterized the uptake and trafficking of wheat germ agglutinin (WGA) during HCMV infection using fluorescence light microscopy and electron microscopy. WGA is a lectin with a high affinity for N-acetylglucosamine and sialic acid, which is internalized into the cell by endocytosis upon binding to glycoproteins on the plasma membrane. WGA has been used to label and to study the endocytic compartment in different cell types, including human fibroblasts, and when coupled with horse radish peroxidase (HRP) allows for visualization of the WGA-positive compartment in electron microscopy [31,32]. In this study, we show that HCMV alters the trafficking of endocytosed WGA compared to uninfected cells, resulting in rapid accumulation of WGA-positive membranes at the cVAC region. In contrast to uninfected cells, the WGA-positive compartment at the cVAC was largely negative for EEA1, indicating viral modulation of endocytic trafficking of cargo beyond early endosomes to provide the membranes for secondary envelopment. WGA-positive membranes at the cVAC, including vesicles and tubules of varying sizes, and trans-most cisternae of Golgi-membrane stacks, were used by nucleocapsids for budding. These findings verified that endocytosed membranes serve as the primary source for the viral envelope. Quantitative analysis of budding capsids at WGA-positive membranes and of enveloped capsids demonstrated that secondary envelopment is a fast and dynamic process. Finally, we found that secondary envelopment of capsids, including endocytosis and transport of membranes, proteins, and capsids to the cVAC, is completed within 30 min, demonstrating a first temporal delineation of these processes in HCMV infection. In conclusion, our results imply extensive modulation of the endocytic compartment by HCMV, which serves as the primary source of membranes used by HCMV for secondary envelopment.

## 2. Materials and Methods

### 2.1. Cell Culture

Human foreskin fibroblasts were cultivated in fibroblast culture medium consisting of Dulbecco’s modified eagle medium (DMEM, Thermo Fisher Scientific Inc., Waltham, MA, USA) supplemented with 100 units/mL penicillin, 100 µg/mL streptomycin, 10% fetal calf serum (FCS), and 1 × non-essential amino acids at 37 °C and 5% CO_2_ in a humidified incubator. Fibroblasts were used between passages 20 and 30 for experiments when a confluency of 90–100% was reached.

### 2.2. Virus

Fibroblasts were infected with HCMV bacterial artificial chromosome (BAC) clone TB40-BAC4 derived from the endotheliotropic HCMV strain TB/40E (accession number EF999921.1 [33]. For all experiments, fibroblasts were infected at a multiplicity of infection (MOI) of 1, resulting in an infection rate of around 60%.

### 2.3. Antibodies

HCMV and cellular proteins were detected in indirect immunofluorescence experiments using mouse monoclonal antibodies directed against cis-Golgi protein marker GM130 (clone 35/GM130, BD Biosciences, Franklin Lakes, NJ, USA), early endosome marker EEA1 (clone 14/EEA1, BD Biosciences), multivesicular body (MVB) marker CD63 (clone H5C6, BD Biosciences), TGN markers Golgin97 (clone CDF4, Thermo Fisher Scientific), γ-Adaptin (clone 100/3, Merck KGaA, Darmstadt, Germany), HCMV pUL99 (clone CH19, Santa Cruz Biotechnology Inc., Dallas, TX, USA), and HCMV gM (IMP 91-31, kindly provided by Michael Mach, University of Erlangen-Nuremberg University, Erlangen, Germany). HCMV pUL71 was detected with a rabbit polyclonal antibody [3]. BrdU was then detected using a rat anti-BrdU monoclonal antibody (RF06, Bio-Rad Laboratories Inc., Hercules, CA, USA) and an anti-rat secondary antibody coupled to Alexa Fluor 555 (Thermo Fisher Scientific). Goat anti-rabbit and goat anti-mouse antibodies conjugated to either Alexa Fluor 555 or Alexa Fluor 647 (Thermo Fisher Scientific) were used as secondary antibodies.

### 2.4. Fluorescence Microscopy

To label endocytic membranes and the recycling compartment in HCMV-infected fibroblasts, we used fluorescence-labeled WGA (WGA-FITC) and Transferrin (Tf-AF555), respectively. Fibroblasts were seeded in µ-Slide 18-well chamber slides (ibidi GmbH, Gräfelfing, Germany) and infected the following day. Pulse-chase labeling was performed at 120 h post-infection (hpi) by incubating the cells with WGA-FITC (10 µg/mL) at 37 °C for 60 min or with WGA-FITC (20 µg/mL) at 4 °C (on ice) for 10 min (pulse). In experiments in which the endocytic and the recycling compartment were labeled, infected fibroblasts were incubated simultaneously with WGA-FITC (10 µg/mL) and Tf-AF555 (50 µg/mL) for 60 min at 37 °C. After that, the labeling solutions were exchanged with fresh fibroblast culture medium. Fibroblasts were then incubated for 30 min at 37 °C (chase). For the endocytosis inhibition control, both pulse and chase were performed at 4 °C (on ice).

Subsequently, the cells were washed with PBS followed by fixation with 4% paraformaldehyde (PFA) in PBS for 10 min at 4 °C. Indirect immunofluorescence staining was performed as previously described [26]. In short, cells were permeabilized with 0.1% Triton X-100 in PBS for 10 min at room temperature followed by the blocking of unspecific binding sites with a blocking solution containing 1% bovine serum albumin and 10% horse serum in PBS for 30 min at room temperature. Primary and secondary antibodies were diluted in a blocking solution. Cell nuclei were stained with 0.33 µg/mL 4,6-diamidino-2-phenylindole (DAPI, Merck KGaA). Representative cells from at least three independent experiments were selected for confocal microscopy. Confocal images were acquired with a 63× objective lens of the Axio-Observer.Z1 fluorescence microscope equipped with an ApoTome2.0 (Zeiss AG, Oberkochen, Germany) and images were processed with the ZEN 3.7 pro software (Zeiss AG).

Nucleocapsids were detected via pulse labeling of newly synthesized DNA with thymidine analog 5-bromo-2′-deoxyuridine (BrdU, Thermo Fisher Scientific). To this end, HCMV-infected fibroblasts were incubated with a 10 µM BrdU solution at 37 °C 24 h before fixation of cells for light microscopy. BrdU detection was performed as described previously [34] with small modifications. Briefly, after permeabilization, cells were treated with 2 M HCl for 15 min at room temperature to expose the incorporated BrdU residues for indirect detection by antibody staining.

### 2.5. WGA-HRP-Labeling and In Vivo DAB-Cytochemistry

To label endocytic membranes in fibroblasts for electron microscopy, pulse-chase labeling with wheat germ agglutinin coupled with horseradish peroxidase (WGA-HRP) was performed, following the protocol established by [32] adapted to HCMV-infected fibroblasts. Briefly, fibroblasts were cultured on carbon-coated sapphire discs (diameter: 3 mm, thickness: 160 µm, Engineering Office M. Wohlwend GmbH, Sennwald, Switzerland) and infected with HCMV or left uninfected. On the day of high-pressure freezing (120 hpi), sapphire discs were transferred to an empty 6-well plate, while the culture medium was removed using filter paper. Subsequently, 10 µL of the WGA-HRP solution (200 µg/mL) in a fibroblast culture medium was directly placed onto each sapphire disc. For the control cells without WGA-HRP labeling but with DAB-H_2_O_2_ treatment, 10 µL of the fibroblast culture medium was added instead. Samples were then incubated for 60 min at 37 °C with 5% CO_2_ or for 10 min at 4 °C (pulse). Then, the WGA-HRP solution was removed from the sapphire discs using filter paper, and the discs were carefully placed into wells of a 6-well plate containing the HEPES-buffered culture medium for 30 min at 37 °C with 5% CO_2_ (chase). For the 4 °C control, this incubation step was performed at 4 °C. To induce the formation of the insoluble 3,3′-diaminobenzidine (DAB) reaction product, the HEPES-buffered culture medium was replaced with a freshly prepared DAB solution (pH 7.0) containing 1.5 mg/mL DAB (Merck KGaA), 70 mM NaCl (Merck KGaA), 50 mM ascorbic acid (VWR International GmbH, Darmstadt, Germany), and 20 mM HEPES buffer. The addition of ascorbic acid as a non-membrane permeable antioxidant prevents the formation of insoluble DAB precipitate on the plasma membrane of the living cell and thus protects the cell from dying. After a 15-min pre-incubation at 4 °C in the dark, H_2_O_2_ (Merck KGaA) was added slowly and dropwise to the DAB solution, which gradually reached the final concentration of 0.025% H_2_O_2_, and cells were incubated for another 30 min at 4 °C in the dark. For the control with WGA-HRP incubation but without the DAB-H_2_O_2_ reaction, cells were transferred to a HEPES-buffered fibroblast culture medium without additives.

### 2.6. Sample Preparation for Electron Microscopy

Samples for electron microscopy were prepared as described in [35] with some modifications. After performing in vivo DAB-cytochemistry, samples were immediately cryo-immobilized in a Wohlwend HPF compact 01 high-pressure freezer (Engineering Office M. Wohlwend GmbH). Samples were subsequently freeze-substituted in a solution containing 1.0% (*v*/*v*) osmium tetroxide (ChemPur, Karlsruhe, Germany) and 0.1% uranyl acetate (*w*/*v*) (Merck KGaA) in acetone, supplemented with 5% (*v*/*v*) water to enhance membrane contrast using the freeze substitution unit EM AFS2 (Leica Microsystems GmbH, Wetzlar, Germany) [36]. Over a period of 17 h, the temperature was gradually increased from −90 °C to 0 °C, with a one-hour incubation step at 0 °C and a further increase to room temperature within one hour. Samples were then rinsed three times with acetone, infiltrated incrementally with Epon 812 (30%, 60%, and 100% Epon in acetone), and finally polymerized at 60 °C for at least 48 h. The sapphire discs were removed, leaving the cells and the carbon coat on the surface of the Epon block.

### 2.7. Transmission Electron Microscopy and Quantification

For transmission electron microscopy (TEM), 70 nm sections were cut from the Epon block using the EM UC7 ultramicrotome (Leica) equipped with a 45° diamond knife (Diatome Ltd., Bern, Switzerland). Sections were collected on freshly glow-discharged single-slot copper grids which were coated with a carbon-reinforced Formvar film. The sections were examined with a JEM-1400 transmission electron microscope (Jeol Ltd., Tokyo, Japan) operating at 120 kV acceleration voltage. Images were acquired using a Veleta CCD camera (Olympus, Tokyo, Japan). The grey value profile was measured for some of the budding and enveloped capsids using the Fiji v2.3.0/1.53q analysis tool “Plot Profile” [37]. At least two independent experiments were performed for capsid quantification. Images were acquired as described previously [38]: Only cVACs containing more than ten capsids were considered for quantification. Images were acquired with sufficient resolution to distinguish the different secondary envelopment stages and WGA labeling and covered the entire cVAC. Capsids were manually classified as budding (WGA-positive or WGA-negative) if they were partially wrapped by a vesicle membrane and if membrane scission had not been completed, e.g., the two arms of the enveloping membrane were still separated. Capsids were classified as enveloped (WGA-positive or WGA-negative) if they were surrounded by a closed viral envelope, forming a spherical virion. Naked capsids (cytoplasmic partially tegumented capsids not in contact with cellular membranes) were excluded from the quantification. The JMicroVision v1.3.4 image analysis software [39] was used for counting capsids in images and the results were visualized using GraphPad 10.1.2.

### 2.8. Scanning Transmission Electron Microscopy Tomography

For three-dimensional (3D) visualization of the Golgi area and budding capsids, scanning transmission electron microscopy (STEM) tomography was performed. For that, samples were prepared as described previously [40]. In brief, 700 nm thick sections were cut from the Epon block using the EM UC7 ultramicrotome (Leica) equipped with a 35° diamond knife. Sections were then collected on freshly glow-discharged copper grids with parallel bars, which were pre-treated with a 10% (*w*/*v*) poly-L-lysine solution (Merck KGaA) in water. After drying for 5 min at 37 °C, sections were immersed in a 1:2 diluted colloidal gold suspension as fiducial markers (AURION, Wageningen, Netherlands) and coated with a 5 nm carbon layer (BAF 300, Balzers, Balzers, Lichtenstein). STEM data were acquired with a JEM-2100F electron microscope (Jeol) operating at 200 kV acceleration voltage and equipped with a bright-field detector (EM-24541SIOD, Jeol). A tilt series of 97 STEM bright-field images was recorded from −72° to 72° at 1.5° increments. Tomograms were reconstructed using the IMOD software package version 4.7 [41] following the procedure described by [42]. The same categories used for quantifying budding and enveloped capsids from TEM images were also used for tomograms. After manual segmentation, 3D visualizations and the video were generated with Avizo 3D 2020.2 (Thermo Fisher Scientific).

## 3. Results

### 3.1. WGA Labels the Endocytic Compartment

**Figure 1:**
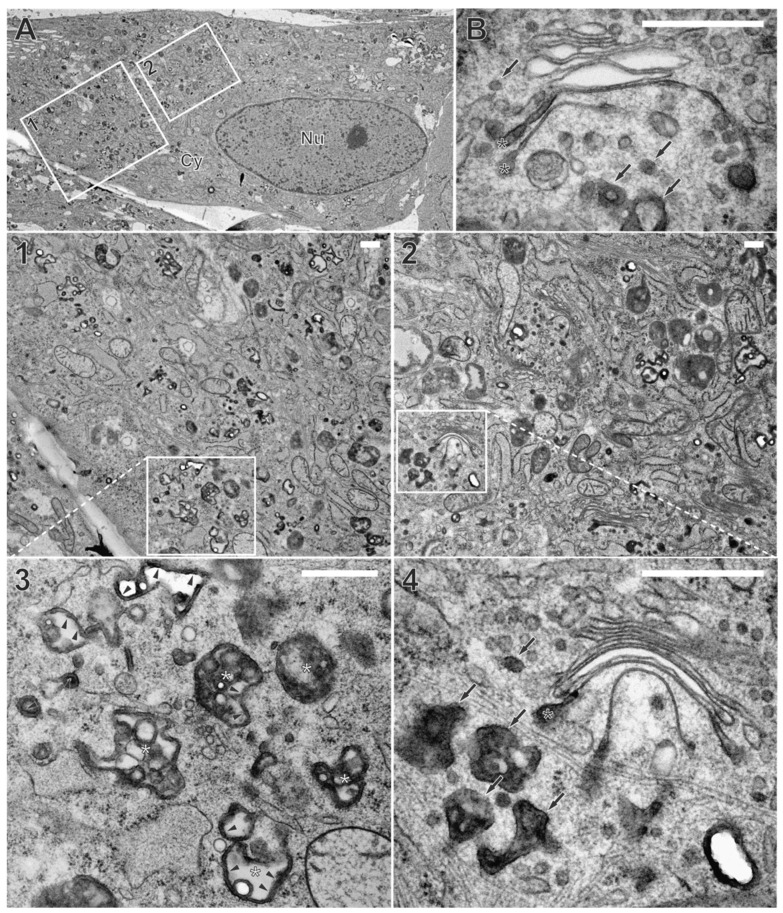
Wheat germ agglutinin (WGA) labels the endocytic compartment. Uninfected fibroblasts were labeled with WGA coupled to horse radish peroxidase (WGA-HRP) for a 60-min pulse and a 30-min chase, both at 37 °C. (**A**) Overview of a cell with corresponding details shown in panels 1–4. (**1**,**2**) DAB precipitate appears dark and labels WGA-positive membranes distributed throughout the cytoplasm. (**3**,**4**) Detailed depiction of DAB precipitate. Note its localization at the luminal face of intercellular membrane compartments (arrowheads). (**3**) Large WGA-positive endosomes are located close to the cell surface, often containing small intraluminal vesicles (=multivesicular bodies (MVBs), white asterisks). (**4**) Vesicles of the endocytic compartment with different sizes (arrows) can be found close to trans-Golgi membranes. WGA-positive membranes were also part of Golgi cisternae (black asterisks), either at the terminal regions of the trans-most Golgi cisternae or (**B**) decorating the entire cross-section of the trans-most cisternae. Cy cytoplasm, Nu nucleus. Scale bars, 500 nm.

We applied WGA-HRP labeling and in vivo DAB-cytochemistry protocols together with high-pressure freezing and freeze substitution (HPF-FS) to identify the endocytic compartment in human fibroblasts by electron microscopy [32]. TEM imaging of human fibroblasts labeled with WGA-HRP showed different intracellular membrane compartments with a luminal electron-dense layer, corresponding to the DAB precipitate (Figure 1A, arrowheads). Such DAB precipitates were not observed in the control cells that were not labeled with WGA-HRP (Figure S1). Thus, internalization and intracellular distribution of WGA-HRP can be detected via these DAB precipitates. Consistent with previous findings [43], the DAB precipitate was frequently concentrated in vesicles of various sizes that were distributed throughout the cytoplasm and are presumably endosomes. In addition, multivesicular bodies (MVBs), appearing as large vesicles with small intraluminal vesicles, were WGA positive (Figure 1A, white asterisks). The WGA-positive endocytic compartment extended to the trans-side of the Golgi apparatus, with DAB precipitate confined either to the entire cross-section of the trans-most Golgi cisternae (Figure 1B, black asterisks) or to their terminal regions (Figure 1A, black asterisks). The observed labeling of trans-Golgi membranes by WGA is consistent with the retrograde transport of proteins and lipids from the plasma membrane via endosomes to the trans-Golgi network (TGN) [44,45]. These results confirm the successful labeling and visualization of the endocytic compartment by WGA-HRP and TEM in human fibroblasts by the applied protocol.

To better define the identity of the WGA-positive membrane compartments observed in TEM, fluorescently labeled WGA (WGA-FITC) was used to label human fibroblasts for 60 min followed by a 30-min chase, both at 37 °C, prior to fixation and indirect immunofluorescence staining for various cellular marker proteins.

**Figure 2:**
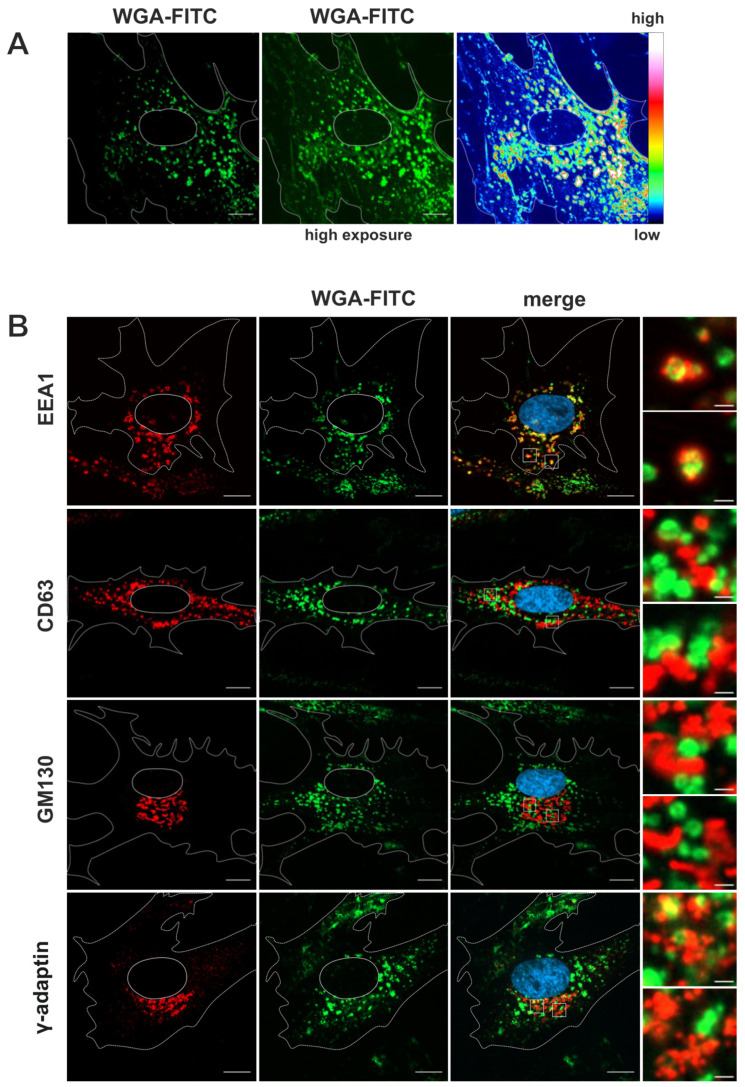
Uptake of WGA-FITC into early endosomes in uninfected cells. Immunofluorescence microscopy of fibroblasts labeled with WGA-FITC (10 µg/mL) with a 60-min pulse and a 30-min chase, both at 37 °C. (**A**) WGA-FITC (green) strongly accumulates in cytoplasmic vesicles of various sizes. Weaker juxtanuclear signals resembling Golgi membranes are also visible (intensity heat map). (**B**) Overlap of WGA-FITC signal with different cellular compartment markers (red). Higher magnification images of selected areas were recorded with longer exposure times to demonstrate the overlap of WGA-positive vesicular signals with EEA1-positive early endosomes. Cell nuclei were stained with DAPI (blue). Scale bars, 10 µm and 1 µm in higher magnifications.

WGA-FITC accumulated in vesicles of various sizes in the cytoplasm (Figure 2A). Most of these signals were perinuclear but also localized near the plasma membrane. Additionally, weaker WGA-FITC signals were observed on juxtanuclear membrane structures that exhibited a similar appearance to Golgi membranes, thereby corroborating the findings of the electron microscopy analysis. Counterstaining for cellular compartment marker proteins by using specific antibodies served to further identify the WGA-positive membrane compartments. EEA1 was used as a marker for early endosomes, CD63 as a marker for MVBs and late endosomes, GM130 as a marker for cis-Golgi membranes [4] and γ-Adaptin [46] (Figure 2B), and Golgin97 [47] as a marker for the TGN (Figure S2). The vesicles that accumulated WGA-FITC were positive for the early endosome marker EEA1, but negative for the MVB marker CD63 as well as negative for TGN and cis-Golgi-markers, confirming the internalization and enrichment of WGA in the early endosomal compartment in human fibroblasts. Together, these experiments demonstrate the rapid internalization and distribution of WGA into the endosomal compartment. In addition, TEM showed further dissemination of WGA to MVBs and trans-Golgi cisternae in uninfected human fibroblasts.

### 3.2. Rapid Accumulation of WGA at the cVAC Together with Nucleocapsids

HCMV infection causes significant changes to intracellular membrane systems, including the endocytic compartment [4,48]. This results in the formation of the cytoplasmic viral assembly complex (cVAC), where nascent virus particles acquire their lipid bilayer envelope through a budding process at intracellular membranes [49]. To investigate HCMV-induced changes in the endocytic compartment and to evaluate its role in cVAC biogenesis and virion maturation, we employed WGA-FITC labeling of HCMV-infected fibroblasts at 120 hpi using a 60-min pulse followed by a 30-min chase.

**Figure 3:**
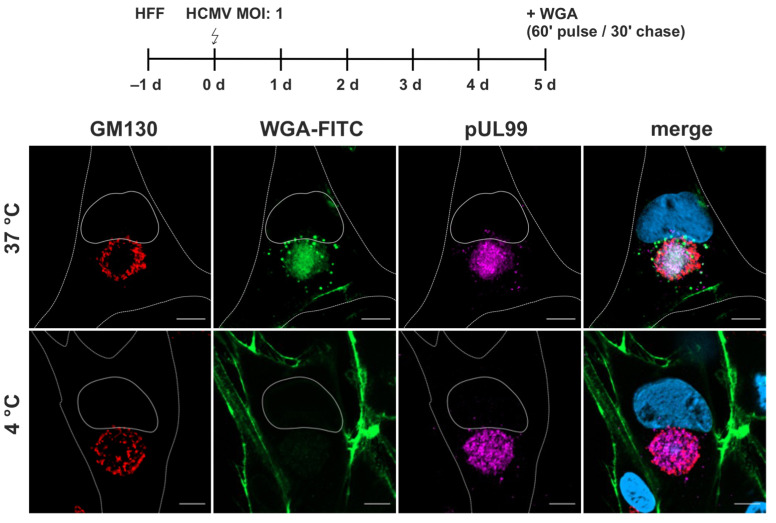
WGA-FITC accumulates at the cVAC in HCMV-infected cells at 120 hpi. Fibroblasts were labeled with WGA-FITC (10 µg/mL) with a 60-min pulse and a 30-min chase, both at either 37 °C or 4 °C. At 37 °C, WGA-FITC (green) is internalized and accumulates as a diffuse signal at the cVAC, which is marked by the viral protein pUL99 (magenta) and delimited by the cis-Golgi marker GM130 (red). In addition, WGA-FITC accumulates in vesicles of different sizes outside the cVAC. Internalization of WGA is inhibited at 4 °C, leading to its accumulation at the plasma membrane. Cell nuclei were stained with DAPI (blue). Scale bars, 10 µm.

WGA-FITC accumulated as a diffuse signal in the region of the cVAC (Figure 3). This region is defined as the juxtanuclear cytoplasmic region positive for the viral tegument protein pUL99, delineated by a ring of cis-Golgi membranes positive for GM130 [7]. The diffuse WGA signals also overlapped with signals for the HCMV tegument protein pUL71 and glycoprotein gM (Figure S3). Both proteins are known to accumulate at the cVAC and play important roles in virion morphogenesis [3,25,26]. In addition to the diffuse signals at the cVAC, WGA-FITC accumulated in vesicles of different sizes surrounding the cVAC. To determine whether the WGA signal at the cVAC resulted from endocytic uptake of WGA at the plasma membrane, WGA labeling was performed under endocytosis-inhibiting conditions at 4 °C [50]. Under these conditions, WGA-FITC was not internalized and remained at the plasma membrane, indicating that accumulation of WGA at the cVAC requires endocytosis of labeled membranes. Together, these results show that a 60-min pulse followed by a 30-min chase at 37 °C is sufficient to concentrate WGA at the cVAC. Furthermore, the difference in WGA distribution compared to uninfected human fibroblasts underscores the extensive re-organization of the cellular membrane systems by HCMV infection.

**Figure 4:**
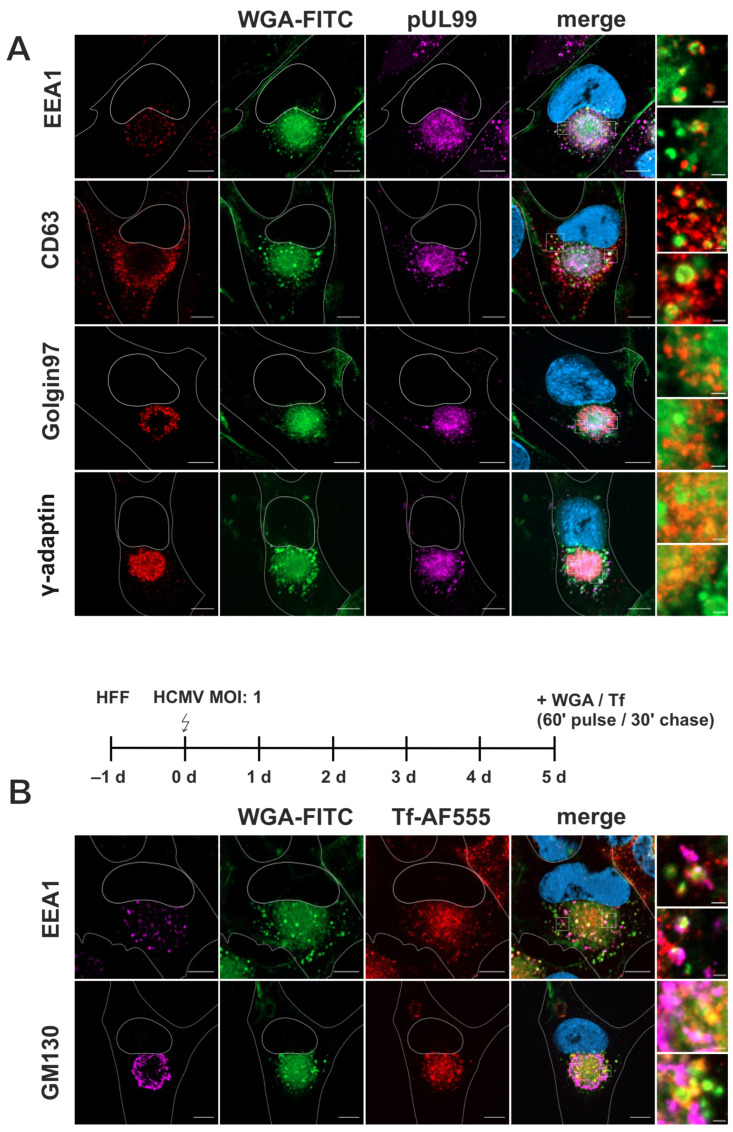
Characterization of the endocytic compartment in HCMV-infected fibroblasts. Higher magnification images were recorded with longer exposure time. (**A**) Screening of cellular compartment markers (red) in relation to WGA-FITC (green) and pUL99 (magenta). Fibroblasts were labeled with WGA-FITC (10 µg/mL) with a 60-min pulse and a 30-min chase, both at 37 °C. Higher magnifications show that the punctate WGA-FITC signals at the periphery of the cVAC overlap with EEA1-positive early endosomes and CD63-positive MVBs. The diffuse WGA-FITC signal within the cVAC exhibits some co-localization with γ-Adaptin. (**B**) Infected fibroblasts were simultaneously labeled with WGA-FITC (10 µg/mL) and Tf-AF555 (50 µg/mL) with a 60-min pulse and a 30-min chase, both at 37 °C. WGA-FITC (green) and Transferrin (Tf, red) localize at the cVAC in a similar pattern. Cellular markers are shown in magenta. Cell nuclei were stained with DAPI (blue). Scale bars, 10 µm and 1 µm in higher magnifications.

As for uninfected fibroblasts, we counterstained WGA-FITC-labeled fibroblasts for cellular organelle markers and examined the overlap of their signals with the signals for WGA-FITC (Figure 4A). Additional staining for HCMV pUL99 served as a marker for infected cells and to indicate the cVAC. EEA1-positive early endosomes were detected surrounding the cVAC and showed overlap with the punctate WGA-FITC signals outside the cVAC region, but not with the diffuse WGA-FITC signals within the cVAC. In contrast to previous data [4], we did not observe the accumulation of EEA1 at the cVAC, suggesting that early endosomes were not enriched at the site of HCMV secondary envelopment in our experimental setup. CD63 signals indicative of MVBs were also observed mainly outside the cVAC region. Some of the CD63-positive vesicles were also positive for WGA-FITC; however, most CD63-positive membranes surrounding the cVAC were negative for WGA-FITC. The partial overlap of WGA with CD63 was not observed in uninfected cells and is an expression of viral manipulation of the cellular membrane systems. As expected, the signal for γ-Adaptin was equally distributed within the cVAC [9,17,51], like pUL99, and exhibited substantial overlap with the diffuse WGA-FITC signals there. In contrast, the TGN-resident membrane marker Golgin97 was found in the peripheral region of the cVAC, thus showing only partial overlap with WGA-FITC at the cVAC.

This data shows a shift in the cellular distribution of WGA-positive membranes upon HCMV infection leading to their accumulation at the cVAC in a diffuse manner, similar to the TGN marker γ-Adaptin. Furthermore, infection causes a shift of WGA-FITC distribution towards MVBs, as more overlap of WGA-FITC signals with those for CD63 is observed in infected than in uninfected fibroblasts (Figure 4).

Several studies have shown a concentration of recycling endosome markers at the cVAC, such as the Transferrin receptor (TfR), Rab11, and Arf6, identifying recycling endosomes as an important structural component of the cVAC [25,52]. To investigate whether the WGA-positive endocytic compartment includes recycling endosomes, we labeled infected fibroblasts simultaneously with WGA-FITC and Transferrin conjugated with AlexaFluor555 (Tf-AF555) for a 60-min pulse followed by a 30-min chase. As shown in Figure 4B, Tf-AF555 accumulated together with WGA-FITC with a similar signal distribution within the cVAC; however, the WGA-FITC signals accumulated in a diffuse, unstructured manner. Consistent with the trafficking of WGA-FITC and TfR through early endosomes [53], several EEA1-positive vesicles surrounding the cVAC were also positive for WGA and Tf-AF555. These data identify recycling endosomes as a component of the endocytic compartment that can be identified by WGA and underscore the potential role of recycling endosomes for cVAC biogenesis and as the potential site of secondary envelopment of HCMV.

**Figure 5:**
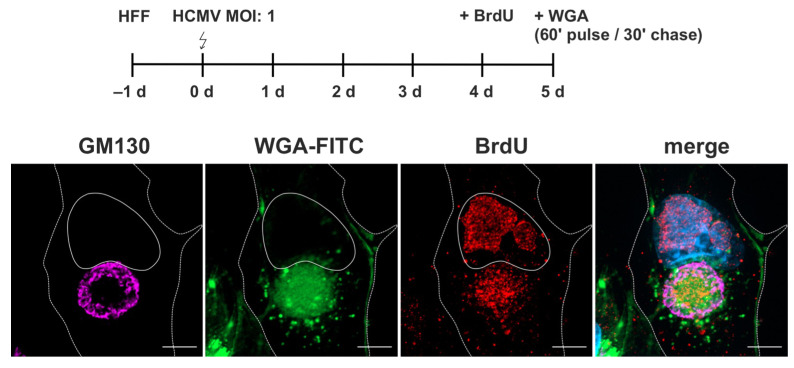
Localization of WGA relative to nucleocapsids. Infected fibroblasts were labeled with a 24-h pulse of BrdU (10 µM) at 96 hpi, followed by WGA-FITC labeling with a 60-min pulse and a 30-min chase at 120 hpi, both at 37 °C. Labeling of viral DNA by BrdU (red) shows the juxtanuclear accumulation of HCMV nucleocapsids at similar regions as the diffuse WGA-FITC signal (green) at the cVAC, here delimited by the GM130 signal (magenta). Cell nuclei were stained with DAPI (blue). Scale bars, 10 µm.

Secondary envelopment requires, in addition to host cell membranes, the presence of viral capsids at the cVAC. To identify nucleocapsids at the cVAC in relation to WGA-positive membranes, we performed BrdU labeling of viral genomes that became incorporated into capsids. The results show an accumulation of BrdU signals at the cVAC, representing nucleocapsids that were generated during the BrdU labeling period. Their signal overlapped with signals of WGA-FITC (Figure 5).

Thus, we could show that labeling of the endocytic compartment by WGA leads to a rapid accumulation of WGA at the cVAC with a distribution pattern like that of viral tegument and glycoproteins and that the WGA-positive endocytic compartment includes membranes of recycling endosomes and the TGN. Furthermore, the accumulation of nucleocapsids at the WGA-positive region suggests that HCMV utilizes WGA-labeled endocytosed membranes for secondary envelopment.

### 3.3. HCMV Utilizes the Endocytic Compartment for Secondary Envelopment

**Figure 6:**
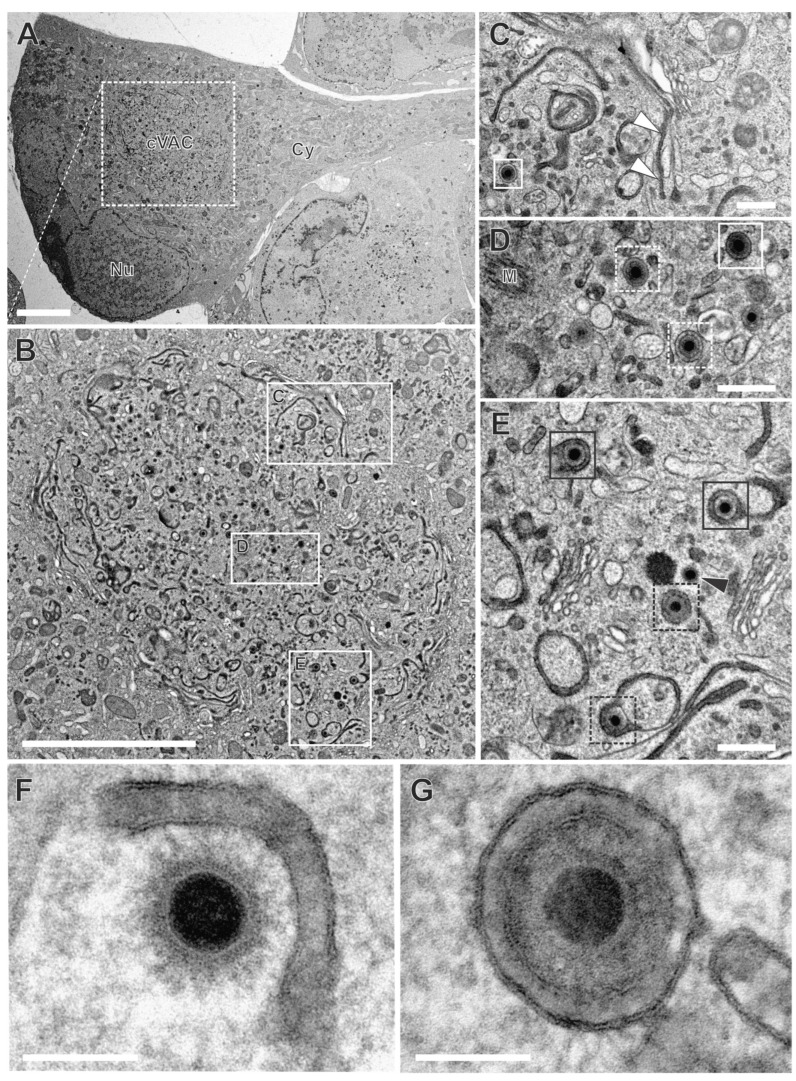
Secondary envelopment at WGA-positive membranes. (**A**) Overview of an HCMV-infected fibroblast labeled with WGA-HRP. Nu nucleus, Cy cytoplasm, cVAC cytoplasmic viral assembly complex. (**B**) WGA-positive membrane compartments within the cVAC, including tubular and spherical vesicles (**B**–**E**) or Golgi cisternae ((**C**), white arrowheads). (**C**–**G**) Capsids at various stages of secondary envelopment and their association with WGA-positive (black boxes) or WGA-negative (white boxes) vesicles. Enveloped capsids (solid boxes), budding capsids (dashed boxes), naked capsid (black arrowhead), M microtubule organizing center. (**F**) Capsid budding into a WGA-positive vesicle and (**G**) enveloped capsid within a WGA-positive vesicle. Quantitative results of this cell in Figure S5B “cell 7”. Scale bars 5 µm (**A**,**B**), 400 nm (**C**,**D**,**E**), 100 nm (**F**,**G**).

To investigate the role of the endocytic compartment in HCMV secondary envelopment, we performed TEM after applying WGA-HRP labeling of HCMV-infected fibroblasts at 120 hpi. The cVACs exhibited conspicuous accumulations of DAB precipitates in the lumen of membranes, indicative of WGA-positive vesicles (Figure 6A,B). These signals were distributed over the entire cVAC region. Vesicles of different sizes and shapes, e.g., tubular and spherical, exhibited characteristic DAB precipitates (Figure 6B–E). Furthermore, the trans-most cisternae of the Golgi stacks were repeatedly found to be WGA-positive (Figure 6C, arrowheads), consistent with our observations in uninfected fibroblasts. It should be noted that not all membranes within the cVAC region were WGA-positive (Figure 6C,D). Importantly, other cellular membranes outside the cVAC, including those of mitochondria, the endoplasmic reticulum, and most Golgi stacks, were lacking DAB precipitates (Figure 6B) and DAB precipitates were absent in infected cells when WGA-HRP labeling was performed under endocytosis-inhibiting conditions at 4 °C (Figure S4) [50]. This verified the requirement of endocytosis for the uptake of WGA into infected cells and confirmed the endocytic origin of the WGA-positive vesicles and the specificity of the WGA labeling. In summary, it was shown that a large proportion of membranes in the cVAC region are provided by the endocytic compartment, thus representing a significant membrane source.

**Figure 7:**
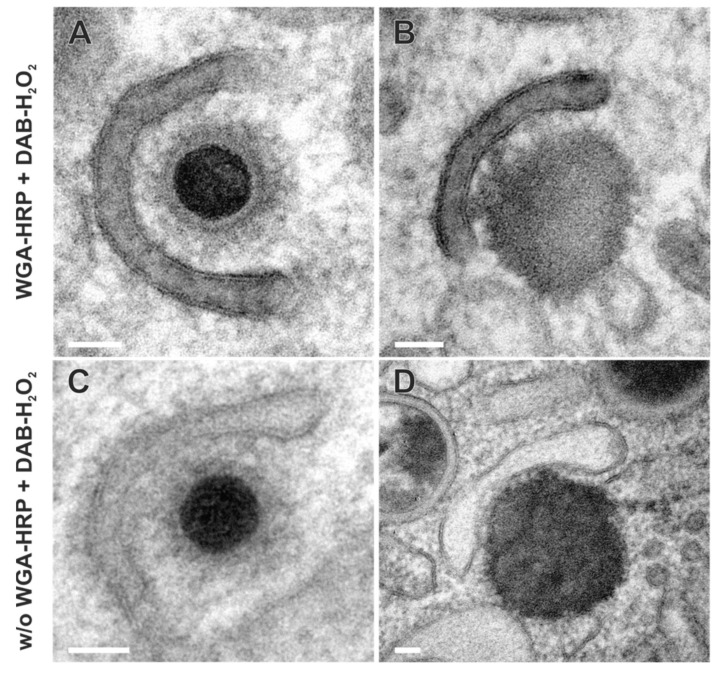
Secondary envelopment of virus particles. Capsid (**A**) and dense body (DB) (**B**) bud into WGA-positive membranes, suggesting a common envelopment mechanism. Capsid (**C**) and DB (**D**) during secondary envelopment in control samples without WGA-HRP labeling. The membrane profiles show no obvious difference when the membranes were labeled with WGA-HRP. Scale bars, 50 nm.

In the cVAC region, the various stages of virion maturation were identified, including naked capsids, capsids undergoing secondary envelopment (budding capsids), and enveloped capsids (Figure 6C–G). These stages are also typically found in infected cells that have not been subjected to the labeling procedure [38,49], demonstrating that WGA labeling has no apparent effect on virus morphogenesis. Many of the membranes on which budding was observed (Figure 6E,F and Figure 7A), as well as membranes of several enveloped capsids (Figure 6E,G), were WGA positive. This clearly shows that membranes of the endocytic compartment are used for secondary envelopment. In addition, as expected, there were also capsids lacking WGA on their membranes (Figure 6D).

In addition to the secondary envelopment of capsids, the formation of dense bodies (DBs) could also be observed in TEM images (Figure 7B,D). DBs are non-infectious virus particles, characterized in TEM as enveloped, electron-dense, homogeneous protein aggregates that are similar in size to virions. In contrast to virions, however, they contain neither a capsid nor an electron-dense DNA core. As for capsids (Figure 7A), the budding of such protein aggregates was observed at WGA-positive membranes (Figure 7B), suggesting a common envelopment mechanism for DBs and capsids. Moreover, the similarity of labeled and non-labeled membrane profiles (Figure 7) indicates no obvious impairment of the envelopment process due to WGA-HRP labeling.

### 3.4. Virion Assembly at the cVAC Is Dynamic

To better understand the extent to which WGA-labeled membranes are involved in secondary envelopment, the different stages of envelopment of HCMV capsids were quantified from TEM images. For that, infected cells were randomly selected for quantification if they exhibited typical morphological features of late-stage infected cells, such as a kidney-shaped nucleus filled with numerous nuclear capsids and recognizable replication centers (Figure 6), accumulations of fragmented ER membranes in the cytoplasm outside the cVAC region [54], and the characteristic cVAC structure delineated by stacked Golgi-cisternae [38]. The capsids in the cVAC region were classified into four categories: 1a labeled (=WGA-positive) budding capsids, 1b non-labeled (=WGA-negative) budding capsids; 2a labeled enveloped capsids and 2b non-labeled enveloped capsids. Unclear virus capsids were classified as WGA-positive or WGA-negative based on a grey-value measurement of the space between the envelope and the vesicle membrane in comparison to the tegument (Figure S5). In the analysis of ten cells, 64.7% of all capsids were classified as budding capsids and 35.3% had completed secondary envelopment (Table 1). A similar ratio of budding versus enveloped capsids was found in cells that were not treated with WGA-HRP, again, confirming that WGA-HRP labeling had no adverse effects on secondary envelopment. When discriminating between budding events at WGA-positive and WGA-negative membranes, it was found that approximately 90% of all budding events occurred at WGA-positive vesicles (Table 1). This indicates that membranes originating from the plasma membrane reach the cVAC within 90 min and that secondary envelopment is initiated during this time. In addition, around 50% of the enveloped capsids were WGA positive, showing that secondary envelopment is not only initiated but also completed within 90 min. Although there were notable differences in the quantification across the 10 cells examined, all cells demonstrated that most capsids were associated with WGA-positive membranes (Figure S5B).

Together, our data demonstrate that secondary envelopment utilizes membranes of the endocytic compartment and indicate that secondary envelopment is a rapid process, highlighting the dynamic nature of HCMV assembly.

To further narrow down the time frame in which the secondary envelopment of HCMV is completed, we modified the labeling procedure. Labeling of late-stage infected fibroblasts was synchronized by their incubation with WGA-HRP at 4 °C for 10 min. The subsequent temperature shift to 37 °C for 30 min allowed the internalization of WGA-HRP into cells. Sufficient internalization and accumulation of WGA at the cVAC by the modified labeling procedure were confirmed using fluorescence microscopy and WGA-FITC (Figure S6).

**Figure 8:**
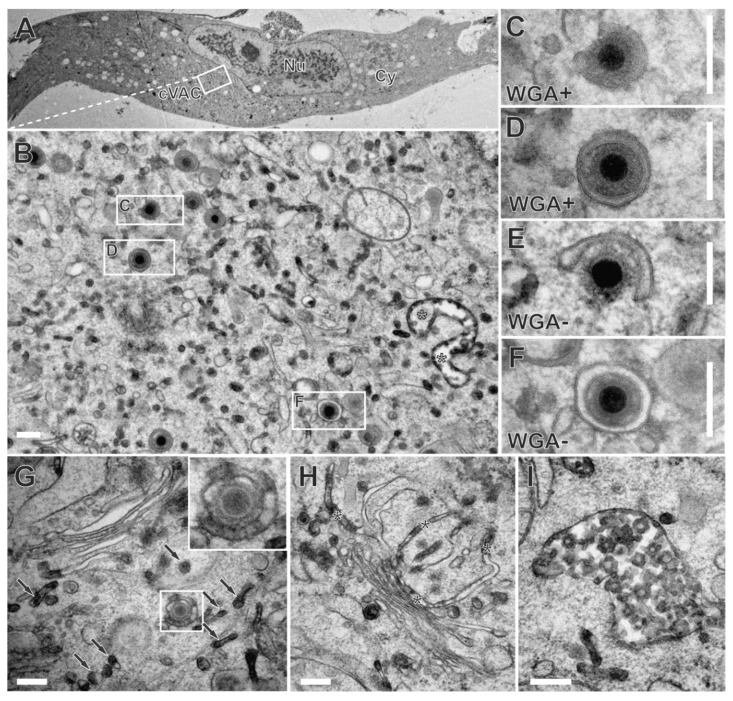
Budding of capsids at WGA-positive membranes as early as 30 min. Infected fibroblast incubated with WGA-HRP for a 10-min pulse at 4 °C and a 30-min chase at 37 °C. (**A**) Overview. Nu nucleus, Cy cytoplasm, cVAC cytoplasmic viral assembly complex. (**B**) A region of the cVAC with WGA-positive large endosomes (white asterisks), and capsids. (**C**) Capsid budding at or (**D**) capsid enveloped in WGA-positive membrane. (**E**) Capsid budding at or (**F**) enveloped in WGA-negative membrane. (**G**) The trans-site of the Golgi apparatus is occupied by multiple WGA-positive vesicles (arrows). Note the budding event at the vesicle with a striped pattern of DAB precipitate (inset). (**H**) Trans-most stacked Golgi cisternae also exhibit a striped pattern of DAB precipitate (black asterisks). (**I**) MVB is filled with numerous WGA-positive intraluminal vesicles. Scale bars, 200 nm.

In electron microscopy, numerous WGA-positive membranes were distributed throughout the cytoplasm of late-stage infected human fibroblasts (Figure 8B). Furthermore, we also observed the budding of capsids at WGA-positive membranes (Figure 8C) and WGA-positive enveloped capsids (Figure 8D). This demonstrates extensive membrane dynamics and indicates that secondary envelopment is completed within a time frame of 30 min. However, as anticipated for a shorter labeling time, budding occurred more frequently at WGA-negative membranes (Figure 8E), and a greater number of WGA-negative enveloped capsids (Figure 8F) was observed in comparison to the experiments with a longer labeling time.

It is noteworthy that the shortening of the labeling pulse resulted in an altered labeling pattern within the cVAC, such that rather small vesicles were WGA-positive (Figure 8B). In addition, the Golgi region exhibited reduced and often less obvious WGA labeling. Compared to the longer labeling time, only one or two of the trans-most Golgi cisternae were visibly WGA-positive. The dark DAB precipitates in those cisternae were limited to their terminal regions or appeared as only partial DAB precipitates distributed in the lumen of the cisternae, leading to a striped pattern in contrast to a lumen that was completely filled with DAB precipitate (Figure 8G,H). This striped pattern was also observed on HCMV envelopes (Figure 8G insert) and may indicate an association of WGA at certain parts of the plasma membrane. Furthermore, MVBs (Figure 8I) and vesicles in the trans-Golgi region were WGA-positive (Figure 8G), confirming the internalization and distribution of WGA along the endocytic pathway as previously reported [55].

### 3.5. Role of the Golgi-Apparatus and the Endocytic Trans-Golgi Network in cVAC Formation and Secondary Envelopment

**Figure 9:**
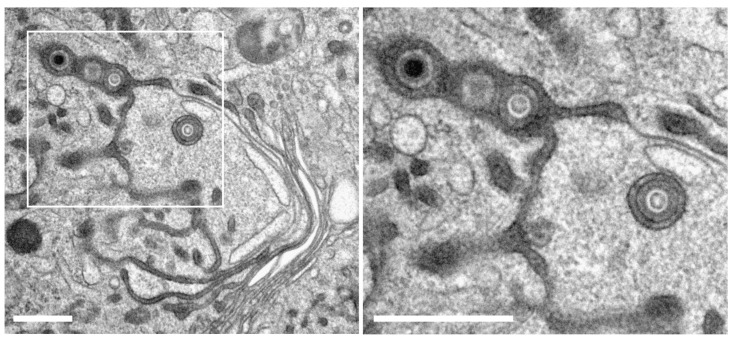
Capsids associated with WGA-positive Golgi membranes. Cells were labeled with WGA-HRP for a 60-min pulse and a 30-min chase. Note the WGA-positive membranes at terminal regions of the trans-sided Golgi cisternae. Scale bar, 500 nm.

It was observed in our experiments that the WGA-positive endocytic compartment in HCMV-infected fibroblasts also included Golgi cisternae. We also found that the trans-side of some of those Golgi stacks contained enveloped capsids (Figure 9). This suggested that the trans-sided membranes of the Golgi stacks can be utilized for secondary envelopment.

We performed three-dimensional (3D) electron microscopy by using scanning transmission electron microscopy (STEM) tomography to examine the structural dependencies between the endocytic compartment, the TGN, and the stacked Golgi cisternae, and to identify their role in HCMV secondary envelopment and cVAC structure. Using tomography, we can unambiguously determine whether capsids have completed the secondary envelopment and whether there is an association of budding capsids with membranes of the trans-side of Golgi stacks. This would confirm secondary envelopment at Golgi membranes. Quantification of secondary envelopment from tomograms confirmed the results from our 2D image quantification, as 68.7% of the capsids were classified as budding and 31.3% were enveloped (Table 2).

**Figure 10:**
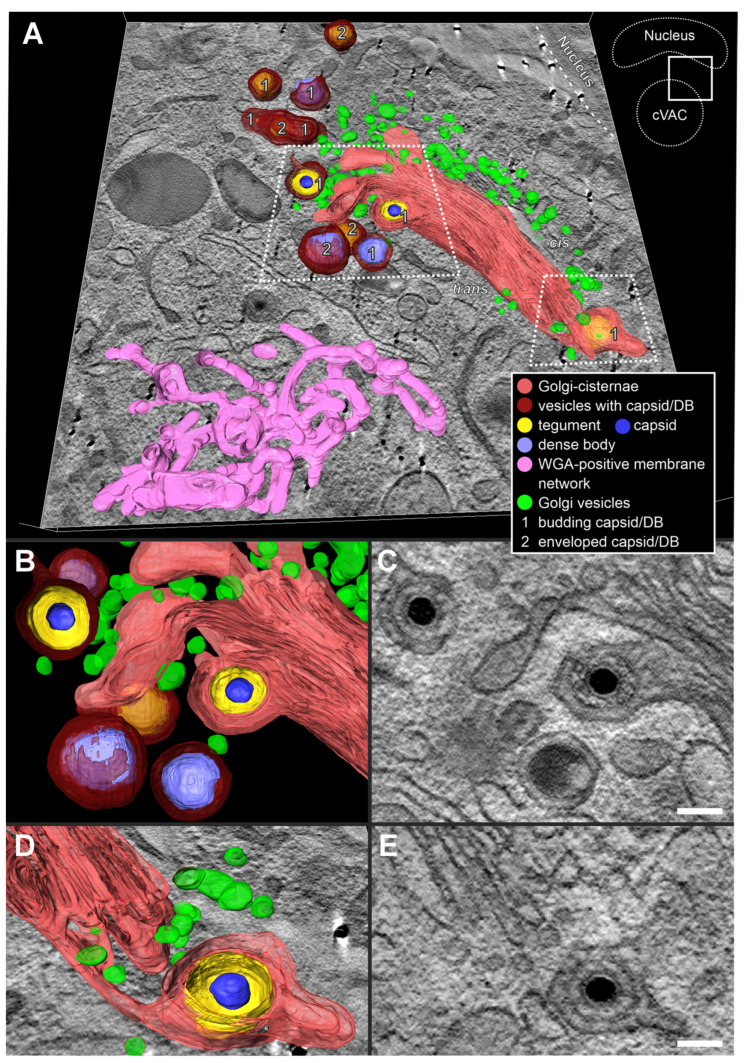
Three-dimensional (3D) visualization of the Golgi region in an HCMV-infected fibroblast. (**A**) Stacked Golgi cisternae with the cis-side facing the nucleus and the trans-side facing the cVAC. Capsids and DBs budding into or enveloped by membranes that are not connected to the Golgi cisternae (dark red) and capsids budding into Golgi cisternae were observed. (**B**–**E**) Capsids budding at the trans-most Golgi cisterna (red). (**D**,**E**) The connection of the budding capsid with the Golgi stack could only be visualized by 3D electron microscopy. Scale bar, 100 nm. Video of this dataset in Supplementary Movie S1.

The tomogram in Figure 10 shows an area of the cVAC that contains a Golgi stack, capsids, and dense bodies at different stages of secondary envelopment. A WGA-positive reticulated structure, similar to what has been described for uninfected cells [55], is located towards the cVAC center (Figure 10A, pink). The cis-side of the Golgi stack faces the nucleus and the trans-side faces the cVAC. Numerous small vesicles, presumably Golgi vesicles, surround the Golgi stack. Several enveloped and budding capsids at membranes not connected to Golgi cisternae are shown. Most interestingly, we found capsids in the process of secondary envelopment at membranes of the trans-most Golgi cisterna (Figure 10B,D; membrane profile visible in Figure 10C,E). The lumen of these Golgi cisternae shows the characteristic staining for WGA-positive membranes, demonstrating its connection to the endocytic compartment. Taken together, our data demonstrate that HCMV utilizes membranes of stacked Golgi cisternae for secondary envelopment and that these membranes are also part of the endocytic compartment.

## 4. Discussion

The general steps of the secondary envelopment of nucleocapsids are similar for all herpesviruses and well understood. Secondary envelopment begins with the binding of partially tegumented nucleocapsids to membranes in the cytoplasm. These contain viral glycoproteins and glycoprotein complexes as well as membrane-associated tegument proteins. The binding of the nucleocapsids to these membranes induces membrane curvature, whereby the membrane wraps around the capsid. The envelopment process is completed by membrane fission, resulting in mature virions located in cytoplasmic vesicles. Although secondary envelopment is an essential process for the formation of infectious viral progeny, little is known about its timing, especially in HCMV morphogenesis. By using WGA to label glycoproteins on the plasma membrane, we were able to narrow down the time frame of secondary envelopment for the first time. In pulse-chase experiments in which we induced the endocytic uptake of WGA into the cell by a temperature shift to 37 °C for 30 min after the binding of WGA to glycoproteins on the plasma membrane, we were able to find budding of nucleocapsids on WGA-positive membranes as well as already enveloped virions in WGA-positive vesicles in the cVAC (Figure 8). This shows that within this time, WGA-labeled glycoproteins are endocytosed and transported from the plasma membrane to the cVAC, where they are recognized by nucleocapsids and secondary envelopment is initiated and also completed. This indicates that the actual time for the secondary envelopment process must be much shorter than 30 min. It could also explain why only a few budding capsids are visible in infected cells while the majority of capsids are already enveloped [49]. Furthermore, our data suggest that HCMV has evolved mechanisms that enable the rapid transport of viral glycoproteins from the plasma membrane to the cVAC and a highly efficient strategy that allows nucleocapsids to recognize the membranes containing the required protein composition for the initiation and execution of secondary envelopment.

Similar studies on HSV-1 using HRP as a fluid phase endocytosis marker have shown that secondary envelopment can be detected at HRP-positive endosomes as early as two minutes after the addition of HRP, and that almost 90% of all capsids were found on HRP-positive membranes with a longer pulse of 30 min [23]. This is similar to our data, confirming that the processes leading to the secondary envelopment of herpesviruses are highly dynamic, but it also indicates that the actual envelopment process of HSV-1 is even more rapid. However, there are some differences between the HSV-1 study and our study that must be considered when comparing the results. Different methodological approaches were used to label the endocytic compartment (fluid phase HRP for HSV-1 versus WGA-HRP in this study). It should also be noted that HSV-1 has a much faster replication cycle than HCMV. In addition, HSV-1 does not establish a cVAC in which the secondary envelopment process takes place, except in certain neuronal cells [56]. However, what both approaches have in common is that the temporal limitation depends on additional processes, such as membrane dynamics, nucleocapsid formation, and intracellular transport of membranes and nucleocapsids to the site of secondary envelopment, which may also be virus and cell-type-dependent.

The cVAC is a highly specialized and dynamic cytoplasmic compartment established by betaherpesviruses [8,9,57]. The cVAC forms around the centrosome, which functions as a microtubule organizing center (MTOC) [2]. In addition, the cVAC itself serves as an MTOC, as HCMV infection induces a switch from centrosomal to non-centrosomal microtubules, which originate at the cis-Golgi membranes of the cVAC [9]. Viral manipulation of the microtubule cytoskeleton is not only crucial for the formation and maintenance of the cVAC [2,9] but is also important for optimized cargo import and export, thus contributing to the highly dynamic envelopment processes during HCMV virion morphogenesis. We show by electron microscopy that a large fraction of the cVAC membranes is WGA-positive after a 60-min pulse followed by a 30-min chase and thus indicate that membranes of the endocytic compartment accumulate at the site of secondary envelopment (Figure 6). Ultrastructural analysis could identify some of these membrane systems as MVBs and trans-most Golgi cisternae. A WGA-positive membrane network, similar to that described in [55], was also identified as part of the cVAC (Figure 10). However, other WGA-positive membranes at the cVAC, especially those serving nucleocapsids for budding, could not be unambiguously identified. Also, counterstaining with various cellular marker proteins, including EEA1 for early endosomes [4], CD63 for late endosomes and MVBs, and Golgi markers, did not result in exact colocalization with the endocytic, WGA-positive compartment (Figure 4). The most similar localization of WGA signals within the pUL99-positive center of the cVAC was found for the trans-Golgi marker γ-Adaptin, and the recycling endosome marker TfR, whereas EEA1-positive early endosomes and late endosomes/MVBs accumulated outside the cis-Golgi signals that mark the boundary of the cVAC. These results and the data from other groups confirm that there is no clear cellular marker for the membranes of the cVAC, especially not for the membranes involved in secondary envelopment. One explanation is that HCMV infection causes extensive changes in membrane compartments and membrane identities [4,48], which is also associated with the manipulation of host cell intracellular transport pathways, including the secretory and endocytic pathways [58,59]. All data to date suggest that HCMV generates the most suitable membrane composition to promote productive infection. This is emphasized by our fluorescence microscopy experiments showing a different distribution of CD63 and WGA in infected fibroblasts compared to uninfected fibroblasts. While WGA did not colocalize with CD63 in uninfected fibroblasts (Figure 2B), some of the CD63-positive vesicles in HCMV-infected fibroblasts were also positive for WGA (Figure 4A). In this work, we show that HCMV nucleocapsids utilize different membranes in the cVAC for secondary envelopment. In addition to the budding of nucleocapsids into endocytic vesicles and tubules of various sizes, budding was also found on membranes of Golgi cisternae (Figure 10). Interestingly, most of these membranes were positive for WGA after a 60-min pulse followed by a 30-min chase, which implies that glycoproteins previously labeled with WGA at the cell surface were retrieved from the plasma membrane and accumulated in these membranes. Thus, the presence of viral glycoproteins appears to be an important aspect of the interaction of nucleocapsids with cellular membranes for the initiation of secondary envelopment. No specific cellular compartment could be assigned to the WGA-positive signals at the cVAC in immunofluorescence analyses with the cellular compartment markers used in this work. However, the budding into different membranes observed by electron microscopy, most of which were WGA-positive, is consistent with all previously postulated models regarding the membrane source of the HCMV envelope [15,16,17] and provides an explanation for these different models. In our experiments, the most common feature of a membrane used for secondary envelopment was that it was a membrane containing glycoproteins that had previously been labeled with WGA on the plasma membrane. WGA-labeled glycoproteins are then distributed through the endocytic compartment and reach other cellular membranes, such as the TGN and trans-sided Golgi cisternae. Although it is important to note that WGA labels both cellular and viral glycoproteins, it is reasonable to assume that a significant proportion of the glycoproteins at the plasma membrane in the late stage of infection are of viral origin. Supporting this, viral glycoproteins are visible in TEM images as patches on the virion envelope [38]. In TEM images of WGA-labeled samples it appeared that the DAB precipitate accumulates preferentially at those patches, forming a striped WGA pattern on the virion envelope (Figure 8G). This model of glycoprotein distribution in HCMV-infected cells is further supported by the presence of endocytic trafficking motifs in the cytoplasmic domain of several viral glycoproteins, such as gB and gM, which play an important role in the localization and accumulation of these proteins at the cVAC. Furthermore, a similar trafficking pathway has already been demonstrated for the membrane-associated tegument protein pUL71 by the characterization of its endocytic motif [26].

We also found mature virions in WGA-negative membranes at the cVAC and also budding at such membranes (Figure 6 and Table 1). We can only speculate about the identity of these membranes. One possible explanation is that these membranes predate the onset of WGA labeling and have not yet been replaced by WGA-labeled membranes. Alternatively, it is also possible that these are membranes originating from other routes and sources or from the chase period. Even if the latter cannot be completely excluded, in fluorescence labeling, a chase of 30 min was not sufficient to purge the plasma membrane of all WGA signals. Future experiments with modified labeling procedures should be able to clarify this. As this example shows, dynamic processes cannot be directly visualized with electron microscopy, as in the end, only snapshots of dynamic events are analyzed. However, with the help of pulse-chase experiments, as performed in this study, we can obtain indirect evidence of the dynamics of the processes investigated here. By this, we were able to get an insight into the dynamics of HCMV morphogenesis. In quantification experiments with a 60-min pulse followed by a 30-min chase, 50% of the capsids that had completed envelopment were WGA-positive (Table 1). This is a remarkably high number considering that at the beginning of the WGA pulse, none of the enveloped capsids in the cVAC are positive for WGA and that several thousand capsids can be present at the cVAC at 120 hpi [49]. Even more remarkable is the finding that about 90% of budding events occurred at WGA-positive membranes. The higher ratio of budding capsids at WGA-positive membranes was expected since budding chronologically precedes full envelopment. Overall, our quantifications indicate that virus particles and membranes in the cVAC are subject to a high turnover and that the WGA-positive compartment at the cVAC is the main membrane source for secondary envelopment of HCMV capsids. It can be assumed that WGA-positive mature virions have already been released from the cells in our labeling experiments. However, due to the methodological procedure of in vivo DAB cytochemistry that requires ascorbic acid in the DAB reaction [32], WGA-positive envelopes cannot be visualized for extracellular virions. Therefore, we can only speculate about the release of labeled virions from the cells. In future studies, visualization of WGA at extracellular virions by alternative means could clarify whether labeled virions are released from infected cells within the labeling period.

The labeling of (viral) glycoproteins at the plasma membrane by WGA led to the labeling of the endocytic compartment. To better understand which membranes are involved in secondary envelopment, future studies could specifically label viral glycoproteins or membrane-associated tegument proteins that have already been shown to be involved in secondary envelopment. This could be conducted through encodable HRP [60,61,62]. Furthermore, more specific labeling of distinct membrane compartments, such as recycling endosomes, could be achieved by using gold-coupled Transferrin.

## 5. Conclusions

In this study, we combined fluorescence microscopy with advanced electron microscopy techniques to investigate the secondary envelopment of HCMV. Taken together, the direct and detailed visualization of capsids in the different stages of secondary envelopment and the high temporal resolution provided by high-pressure freezing, combined with the labeling of the endocytic compartment, revealed that endocytosed membranes are the major source for the cVAC membranes and for the HCMV envelope, which is acquired in a rapid process.

## Figures and Tables

**Table 1:** Quantification of budding and enveloped capsids and their association with WGA-positive (WGA+) and WGA-negative (WGA-) membranes from TEM images of HCMV infected human fibroblasts (120 hpi) treated with WGA-HRP (60-min pulse, 30-min chase) followed by DAB and of cells not treated with WGA-HRP but with DAB only from one representative experiment. Given are the mean percentages of budding and enveloped capsids per cVAC with standard deviation (SD), absolute numbers in brackets. The total number of budding and enveloped capsids per cell, respectively, was set to 100% to determine the proportion of WGA-positive capsids among these categories.

Sample	Membrane	Mean % ± SD (No. of Capsids)	
		Budding	Enveloped
With WGA-HRP (423 capsids, 10 cells)		64.7% ± 9.5 (265)	35.3% ± 9.5 (158)
	WGA-pos.	**88.0%** ± 9.2 (227)	**48.8**% ± 19.0 (72)
	WGA-neg.	12.0% ± 9.2 (38)	51.2% ± 19.0 (86)
Without WGA-HRP (291 capsids, 9 cells)		63.2% ± 7.8 (183)	36.8% ± 7.8 (108)

**Table 2:** Quantification of budding and enveloped capsids in human fibroblasts treated with WGA-HRP (60-min pulse, 30-min chase). Shown are the mean percentages of budding and enveloped capsids from five different STEM tomograms of random cVAC areas. Standard deviation (SD), absolute numbers in brackets.

Sample	Mean % ± SD (No. of Capsids)	
	Budding	Enveloped
With WGA-HRP (69 capsids, 5 tomograms)	68.7% ± 10.8 (47)	31.3% ± 10.8 (22)

## Data Availability

Raw data is available upon request.

## Supplementary Materials

The following supporting information can be downloaded at: https://www.mdpi.com/article/10.3390/biom14091149/s1, Supplementary Movie S1: 3D visualization of the Golgi region of an HCMV-infected fibroblast; Figure S1: Uninfected fibroblast not labeled with WGA-HRP but treated with DAB and H_2_O_2_; Figure S2: Colocalization study of TGN protein markers and WGA-FITC in uninfected fibroblasts; Figure S3: Immunofluorescence microscopy of viral proteins pUL71 and gM and WGA-FITC at the cVAC in HCMV-infected fibroblasts at 120 hpi; Figure S4: WGA-internalization is inhibited at 4 °C; Figure S5: Quantification of secondary envelopment at WGA-positive and WGA-negative membranes; Figure S6: Internalization of WGA-FITC in HCMV-infected fibroblasts at 120 hpi after shorter labeling times.

## References

[1] Das S., Vasanji A., Pellett P.E. (2007). Three-Dimensional Structure of the Human Cytomegalovirus Cytoplasmic Virion Assembly Complex Includes a Reoriented Secretory Apparatus. J. Virol..

[2] Sanchez V., Greis K.D., Sztul E., Britt W.J. (2000). Accumulation of Virion Tegument and Envelope Proteins in a Stable Cytoplasmic Compartment during Human Cytomegalovirus Replication: Characterization of a Potential Site of Virus Assembly. J. Virol..

[3] Schauflinger M., Fischer D., Schreiber A., Chevillotte M., Walther P., Mertens T., von Einem J. (2011). The Tegument Protein UL71 of Human Cytomegalovirus Is Involved in Late Envelopment and Affects Multivesicular Bodies. J. Virol..

[4] Das S., Pellett P.E. (2011). Spatial Relationships between Markers for Secretory and Endosomal Machinery in Human Cytomegalovirus-Infected Cells versus Those in Uninfected Cells. J. Virol..

[5] Fraile-Ramos A., Kledal T.N., Pelchen-Matthews A., Bowers K., Schwartz T.W., Marsh M. (2001). The Human Cytomegalovirus US28 Protein Is Located in Endocytic Vesicles and Undergoes Constitutive Endocytosis and Recycling. Mol. Biol. Cell.

[6] Fraile-Ramos A., Pelchen-Matthews A., Kledal T.N., Browne H., Schwartz T.W., Marsh M. (2002). Localization of HCMV UL33 and US27 in Endocytic Compartments and Viral Membranes. Traffic.

[7] Das S., Ortiz D.A., Gurczynski S.J., Khan F., Pellett P.E. (2014). Identification of Human Cytomegalovirus Genes Important for Biogenesis of the Cytoplasmic Virion Assembly Complex. J. Virol..

[8] Lučin P., Jug Vučko N., Karleuša L., Mahmutefendić Lučin H., Blagojević Zagorac G., Lisnić B., Pavišić V., Marcelić M., Grabušić K., Brizić I. (2020). Cytomegalovirus Generates Assembly Compartment in the Early Phase of Infection by Perturbation of Host-Cell Factors Recruitment at the Early Endosome/Endosomal Recycling Compartment/Trans-Golgi Interface. Front. Cell Dev. Biol..

[9] Procter D.J., Banerjee A., Nukui M., Kruse K., Gaponenko V., Murphy E.A., Komarova Y., Walsh D. (2018). The HCMV Assembly Compartment Is a Dynamic Golgi-Derived MTOC That Controls Nuclear Rotation and Virus Spread. Dev. Cell.

[10] Wofford A.S., McCusker I., Green J.C., Vensko T.A., Pellett P.E., Kielian M., Mettenleiter T.C., Roossinck M.J. (2020). Chapter Ten—Betaherpesvirus Assembly and Egress: Recent Advances Illuminate the Path. Advances in Virus Research.

[11] Cruz L., Streck N.T., Ferguson K., Desai T., Desai D.H., Amin S.G., Buchkovich N.J. (2016). Potent Inhibition of Human Cytomegalovirus by Modulation of Cellular SNARE Syntaxin 5. J. Virol..

[12] Varnum S.M., Streblow D.N., Monroe M.E., Smith P., Auberry K.J., Pasa-Tolic L., Wang D., Camp D.G., Rodland K., Wiley S. (2004). Identification of Proteins in Human Cytomegalovirus (HCMV) Particles: The HCMV Proteome. J. Virol..

[13] Liu F., Zhou Z.H., Arvin A., Campadelli-Fiume G., Mocarski E., Moore P.S., Roizman B., Whitley R., Yamanishi K. (2007). Comparative Virion Structures of Human Herpesviruses. Human Herpesviruses: Biology, Therapy, and Immunoprophylaxis.

[14] Kalejta R.F. (2008). Tegument Proteins of Human Cytomegalovirus. Microbiol. Mol. Biol. Rev..

[15] Tooze J., Hollinshead M., Reis B., Radsak K., Kern H. (1993). Progeny Vaccinia and Human Cytomegalovirus Particles Utilize Early Endosomal Cisternae for Their Envelopes. Eur. J. Cell Biol..

[16] Homman-Loudiyi M., Hultenby K., Britt W., Soderberg-Naucler C. (2003). Envelopment of Human Cytomegalovirus Occurs by Budding into Golgi-Derived Vacuole Compartments Positive for gB, Rab 3, Trans-Golgi Network 46, and Mannosidase II. J. Virol..

[17] Cepeda V., Esteban M., Fraile-Ramos A. (2010). Human Cytomegalovirus Final Envelopment on Membranes Containing Both Trans -Golgi Network and Endosomal Markers. Cell. Microbiol..

[18] Gershon A.A., Sherman D.L., Zhu Z., Gabel C.A., Ambron R.T., Gershon M.D. (1994). Intracellular Transport of Newly Synthesized Varicella-Zoster Virus: Final Envelopment in the Trans-Golgi Network. J. Virol..

[19] Granzow H., Weiland F., Jöns A., Klupp B.G., Karger A., Mettenleiter T.C. (1997). Ultrastructural Analysis of the Replication Cycle of Pseudorabies Virus in Cell Culture: A Reassessment. J. Virol..

[20] McMillan T.N., Johnson D.C. (2001). Cytoplasmic Domain of Herpes Simplex Virus gE Causes Accumulation in the Trans-Golgi Network, a Site of Virus Envelopment and Sorting of Virions to Cell Junctions. J. Virol..

[21] Whiteley A., Bruun B., Minson T., Browne H. (1999). Effects of Targeting Herpes Simplex Virus Type 1 gD to the Endoplasmic Reticulum and Trans-Golgi Network. J. Virol..

[22] Zhu Z., Gershon M.D., Hao Y., Ambron R.T., Gabel C.A., Gershon A.A. (1995). Envelopment of Varicella-Zoster Virus: Targeting of Viral Glycoproteins to the Trans-Golgi Network. J. Virol..

[23] Hollinshead M., Johns H.L., Sayers C.L., Gonzalez-Lopez C., Smith G.L., Elliott G. (2012). Endocytic Tubules Regulated by Rab GTPases 5 and 11 Are Used for Envelopment of Herpes Simplex Virus. EMBO J..

[24] Radsak K., Eickmann M., Mockenhaupt T., Bogner E., Kern H., Eis-Hübinger A., Reschke M. (1996). Retrieval of Human Cytomegalovirus Glycoprotein B from the Infected Cell Surface for Virus Envelopment. Arch. Virol..

[25] Krzyzaniak M.A., Mach M., Britt W.J. (2009). HCMV-Encoded Glycoprotein M (UL100) Interacts with Rab11 Effector Protein FIP4. Traffic.

[26] Dietz A.N., Villinger C., Becker S., Frick M., Von Einem J. (2018). A Tyrosine-Based Trafficking Motif of the Tegument Protein pUL71 Is Crucial for Human Cytomegalovirus Secondary Envelopment. J. Virol..

[27] Tugizov S., Maidji E., Xiao J., Pereira L. (1999). An Acidic Cluster in the Cytosolic Domain of Human Cytomegalovirus Glycoprotein B Is a Signal for Endocytosis from the Plasma Membrane. J. Virol..

[28] Archer M.A., Brechtel T.M., Davis L.E., Parmar R.C., Hasan M.H., Tandon R. (2017). Inhibition of Endocytic Pathways Impacts Cytomegalovirus Maturation. Sci. Rep..

[29] Štimac I., Jug Vučko N., Blagojević Zagorac G., Marcelić M., Mahmutefendić Lučin H., Lučin P. (2021). Dynamin Inhibitors Prevent the Establishment of the Cytomegalovirus Assembly Compartment in the Early Phase of Infection. Life.

[30] Hasan M.H., Davis L.E., Bollavarapu R.K., Mitra D., Parmar R., Tandon R. (2018). Dynamin Is Required for Efficient Cytomegalovirus Maturation and Envelopment. J. Virol..

[31] Pavelka M., Ellinger A., Debbage P., Loewe C., Vetterlein M., Roth J. (1998). Endocytic Routes to the Golgi Apparatus. Histochemistry.

[32] Ellinger A., Vetterlein M., Weiss C., Meißlitzer-Ruppitsch C., Neumüller J., Pavelka M. (2010). High-Pressure Freezing Combined with In Vivo-DAB-Cytochemistry: A Novel Approach for Studies of Endocytic Compartments. J. Struct. Biol..

[33] Sinzger C., Hahn G., Digel M., Katona R., Sampaio K.L., Messerle M., Hengel H., Koszinowski U., Brune W., Adler B. (2008). Cloning and Sequencing of a Highly Productive, Endotheliotropic Virus Strain Derived from Human Cytomegalovirus TB40/E. J. Gen. Virol..

[34] Rosenke K., Fortunato E.A. (2004). Bromodeoxyuridine-Labeled Viral Particles as a Tool for Visualization of the Immediate-Early Events of Human Cytomegalovirus Infection. J. Virol..

[35] Read C., Schauflinger M., Nikolaenko D., Walther P., von Einem J. (2019). Regulation of Human Cytomegalovirus Secondary Envelopment by a C-Terminal Tetralysine Motif in pUL71. J. Virol..

[36] Walther P., Ziegler A. (2002). Freeze Substitution of High-Pressure Frozen Samples: The Visibility of Biological Membranes Is Improved When the Substitution Medium Contains Water. J. Microsc..

[37] Schindelin J., Arganda-Carreras I., Frise E., Kaynig V., Longair M., Pietzsch T., Preibisch S., Rueden C., Saalfeld S., Schmid B. (2012). Fiji: An Open-Source Platform for Biological-Image Analysis. Nat. Methods.

[38] Read C., Walther P., von Einem J., Yurochko A.D. (2021). Quantitative Electron Microscopy to Study HCMV Morphogenesis. Human Cytomegaloviruses: Methods and Protocols.

[39] Roduit N. JMicroVision: Image Analysis Toolbox for Measuring and Quantifying Components of High-Definition Images. Version 1.3.4. https://jmicrovision.github.io.

[40] Bergner T., Zech F., Hirschenberger M., Stenger S., Sparrer K.M.J., Kirchhoff F., Read C. (2022). Near-Native Visualization of SARS-CoV-2 Induced Membrane Remodeling and Virion Morphogenesis. Viruses.

[41] Kremer J.R., Mastronarde D.N., McIntosh J.R. (1996). Computer Visualization of Three-Dimensional Image Data Using IMOD. J. Struct. Biol..

[42] Wieland J., Frey S., Rupp U., Essbauer S., Groß R., Münch J., Walther P. (2021). Zika Virus Replication in Glioblastoma Cells: Electron Microscopic Tomography Shows 3D Arrangement of Endoplasmic Reticulum, Replication Organelles, and Viral Ribonucleoproteins. Histochem. Cell Biol..

[43] Ranftler C., Auinger P., Meisslitzer-Ruppitsch C., Ellinger A., Neumüller J., Pavelka M., Taatjes D.J., Roth J. (2013). Electron Microscopy of Endocytic Pathways. Cell Imaging Techniques: Methods and Protocols.

[44] Tu Y., Zhao L., Billadeau D.D., Jia D. (2020). Endosome-to-TGN Trafficking: Organelle-Vesicle and Organelle-Organelle Interactions. Front. Cell Dev. Biol..

[45] Pavelka M., Neumüller J., Ellinger A. (2008). Retrograde Traffic in the Biosynthetic-Secretory Route. Histochem. Cell Biol..

[46] Wang X., Cai Y., Wang H., Zeng Y., Zhuang X., Li B., Jiang L. (2014). Trans-Golgi Network-Located AP1 Gamma Adaptins Mediate Dileucine Motif-Directed Vacuolar Targeting in *Arabidopsis*. Plant Cell.

[47] Lu L., Tai G., Hong W. (2004). Autoantigen Golgin-97, an Effector of Arl1 GTPase, Participates in Traffic from the Endosome to the Trans-Golgi Network. Mol. Biol. Cell.

[48] Jean Beltran P.M., Mathias R.A., Cristea I.M. (2016). A Portrait of the Human Organelle Proteome in Space and Time during Cytomegalovirus Infection. Cell Syst..

[49] Schauflinger M., Villinger C., Mertens T., Walther P., von Einem J. (2013). Analysis of Human Cytomegalovirus Secondary Envelopment by Advanced Electron Microscopy. Cell. Microbiol..

[50] Breton S., Brown D. (1998). Cold-Induced Microtubule Disruption and Relocalization of Membrane Proteins in Kidney Epithelial Cells. J. Am. Soc. Nephrol..

[51] Jarvis M.A., Fish K.N., Söderberg-Naucler C., Streblow D.N., Meyers H.L., Thomas G., Nelson J.A. (2002). Retrieval of Human Cytomegalovirus Glycoprotein B from Cell Surface Is Not Required for Virus Envelopment in Astrocytoma Cells. J. Virol..

[52] Lučin P., Kareluša L., Blagojević Zagorac G., Mahmutefendić Lučin H., Pavišić V., Jug Vučko N., Lukanović Jurić S., Marcelić M., Lisnić B., Jonjić S. (2018). Cytomegaloviruses Exploit Recycling Rab Proteins in the Sequential Establishment of the Assembly Compartment. Front. Cell Dev. Biol..

[53] Mayle K.M., Le A.M., Kamei D.T. (2012). The Intracellular Trafficking Pathway of Transferrin. Biochim. Biophys. Acta.

[54] Zhang H., Read C., Nguyen C.C., Siddiquey M.N.A., Shang C., Hall C.M., von Einem J., Kamil J.P. (2019). The Human Cytomegalovirus Nonstructural Glycoprotein UL148 Reorganizes the Endoplasmic Reticulum. mBio.

[55] Vetterlein M., Ellinger A., Neumüller J., Pavelka M. (2002). Golgi Apparatus and TGN during Endocytosis. Histochem. Cell Biol..

[56] White S., Roller R. (2024). Herpes simplex virus type-1 cVAC formation in neuronal cells is mediated by dynein motor function and glycoprotein retrieval from the plasma membrane. J. Virol..

[57] Alwine J.C. (2012). The Human Cytomegalovirus Assembly Compartment: A Masterpiece of Viral Manipulation of Cellular Processes That Facilitates Assembly and Egress. PLOS Pathog..

[58] Hook L.M., Grey F., Grabski R., Tirabassi R., Doyle T., Hancock M., Landais I., Jeng S., McWeeney S., Britt W. (2014). Cytomegalovirus miRNAs Target Secretory Pathway Genes to Facilitate Formation of the Virion Assembly Compartment and Reduce Cytokine Secretion. Cell Host Microbe.

[59] Turner D.L., Mathias R.A. (2022). The Human Cytomegalovirus Decathlon: Ten Critical Replication Events Provide Opportunities for Restriction. Front. Cell Dev. Biol..

[60] Joesch M., Mankus D., Yamagata M., Shahbazi A., Schalek R., Suissa-Peleg A., Meister M., Lichtman J.W., Scheirer W.J., Sanes J.R. (2016). Reconstruction of Genetically Identified Neurons Imaged by Serial-Section Electron Microscopy. eLife.

[61] Sengupta R., Poderycki M.J., Mattoo S. (2019). CryoAPEX—An Electron Tomography Tool for Subcellular Localization of Membrane Proteins. J. Cell Sci..

[62] Tsang T.K., Bushong E.A., Boassa D., Hu J., Romoli B., Phan S., Dulcis D., Su C.-Y., Ellisman M.H. (2018). High-Quality Ultrastructural Preservation Using Cryofixation for 3D Electron Microscopy of Genetically Labeled Tissues. eLife.

