# Supplementary material for: Secondary Envelopment of Human Cytomegalovirus Is a Fast Process Utilizing the Endocytic Compartment as a Major Membrane Source
Source: Biomolecules. 2024 Sep 12;14(9):1149. doi: 10.3390/biom14091149 (PMC11430300; doi:10.3390/biom14091149)
Supplement: Supplementary file 1 [file biomolecules-14-01149-s001.zip › Supplementary_biomolecules-3175511_proof.pdf]

Supplementary files

Supplementary Movie 1: 3D visualization of the Golgi region of an HCMV-infected fibroblast. Dataset from Figure 10. STEM-tomogram of 150 virtual tomography sections resulting in a thickness of 450 nm showing stacked Golgi cisternae (light red), surrounding Golgi vesicles (green) and a WGA-positive interconnected membrane network (pink). Some capsids (blue, with yellow tegument) and DBs (purple) are associated with membranes that are not connected to the Golgi stack (dark red). The movie of the 3D dataset showcases budding of viral capsids at the trans-most Golgi cisterna.

Figure S1

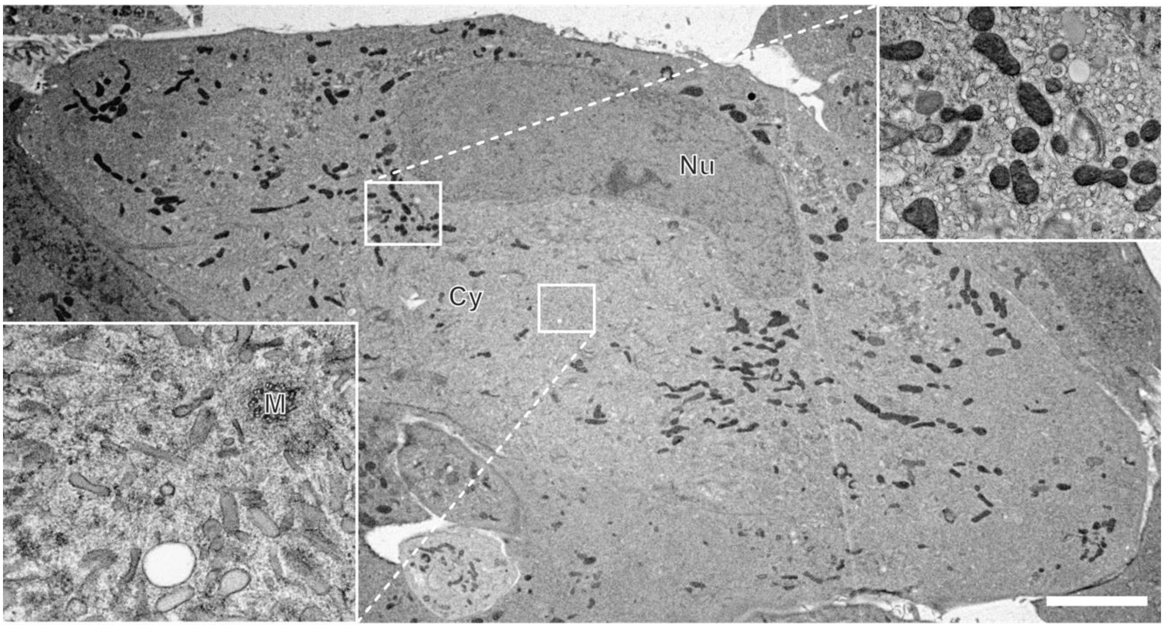

Figure S1: Uninfected fibroblast not labelled with WGA-HRP but treated with DAB and H_2O_2. No DAB precipitate is visible within the cytoplasm. Nu nucleus, Cy cytoplasm, M MTOC. Right inset shows that the dark structures in the cytoplasm represent mitochondria. Scale bar, 5 µm.

Figure S2

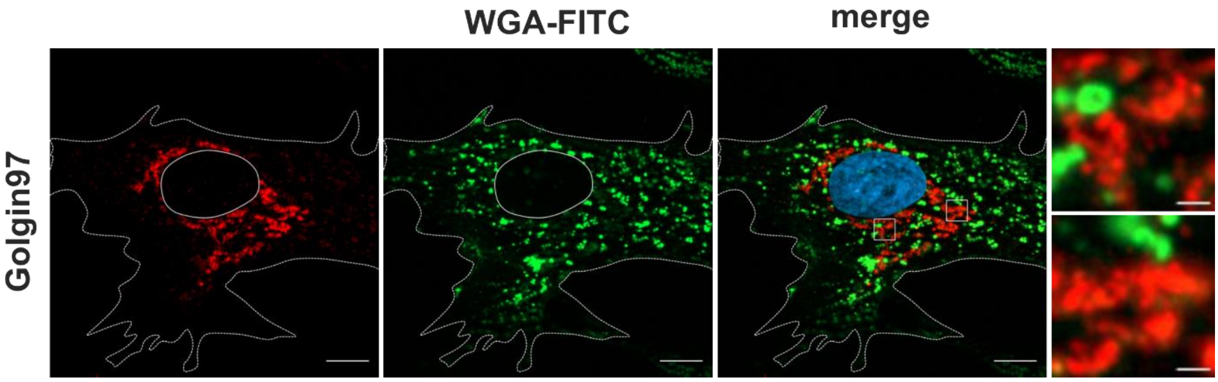

Figure S2: Colocalization study of TGN protein marker Golgin97 and WGA-FITC in uninfected fibroblasts. After 60 minutes pulse and 30 minutes chase, both at 37°C, WGA-FITC (green) and Golgin97 (red) are not overlapping. Cell nuclei were stained with DAPI (blue). Scale bars, 10 µm and 1 µm in higher magnifications.

Figure S3

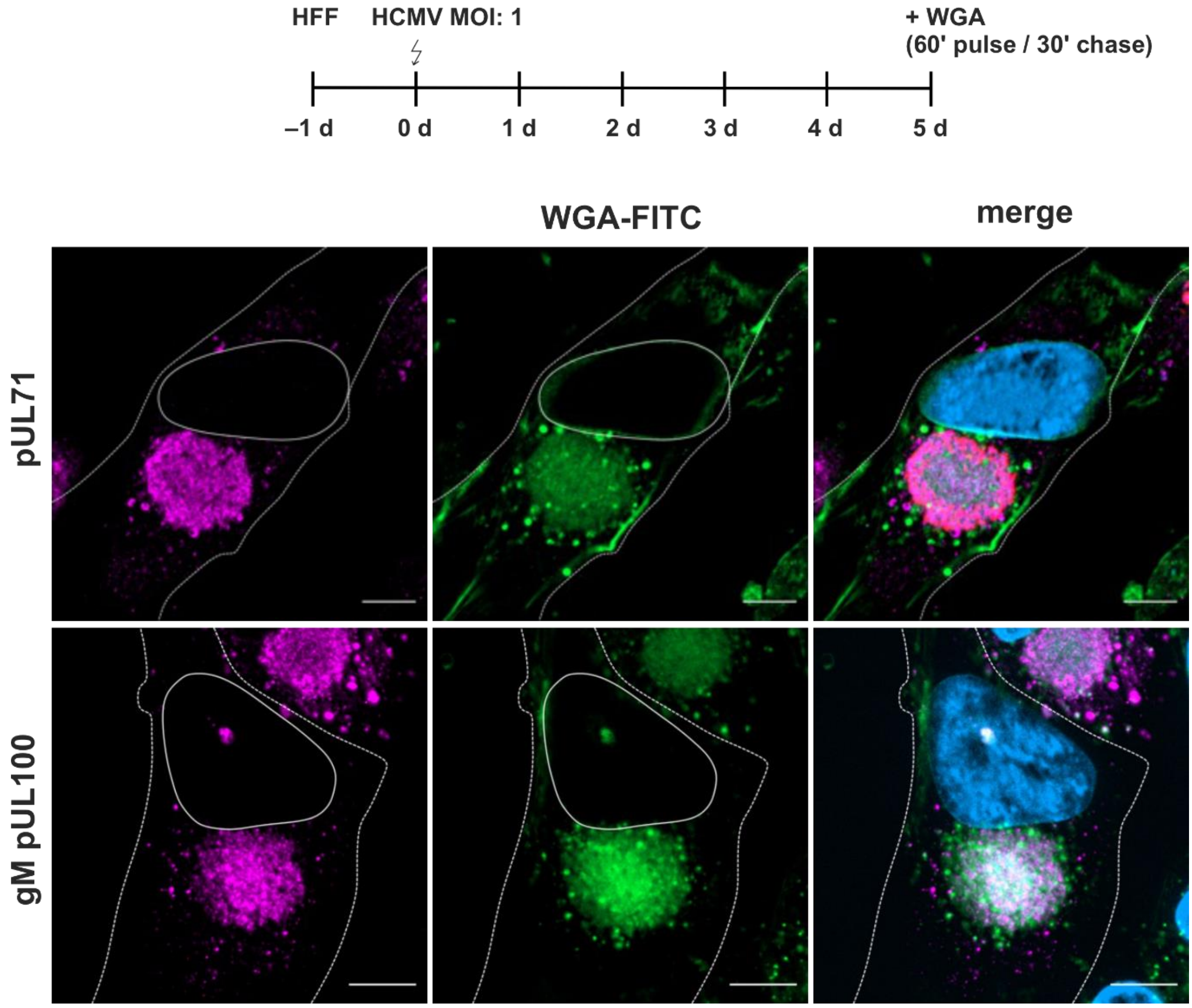

Figure S3: Immunofluorescence microscopy of viral proteins pUL71 and gM (magenta) and WGA-FITC (green) at the cVAC in HCMV-infected fibroblasts at 120 hpi. Infected fibroblasts were labelled with WGA-FITC (10 µg/ml) with a 60 minutes pulse and 30 minutes chase, both at 37°C. WGA accumulates at the cVAC. Cell nuclei were stained with DAPI (blue). Scale bars, 10 µm.

Figure S4

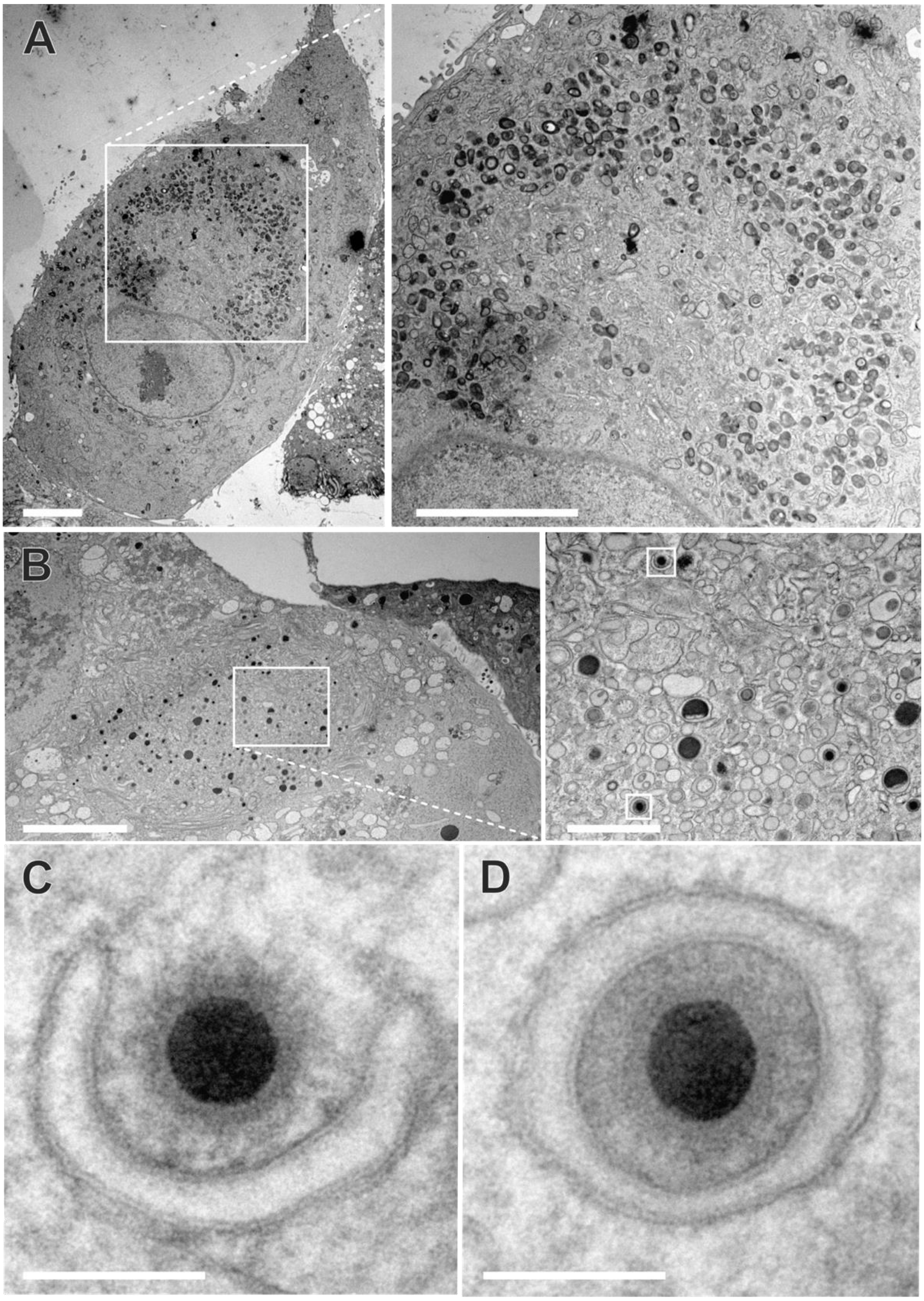

Figure S4: WGA-internalization is inhibited at 4°C. Uninfected (A) and HCMV-infected (B) fibroblasts, incubated with WGA-HRP for a 60 minutes pulse and 30 minutes chase, both at 4°C, and subjected to *in vivo* DAB cytochemistry. As the internalization of WGA-HRP is reduced at 4°C, no WGA-labelled membranes are present in the cytoplasm. Budding (C) and enveloped capsids (D) assoiated with WGA-negative membranes. Scale bars 5 µm (A and B left), 1 µm (B right) and 100 nm (C and D).

Figure S5

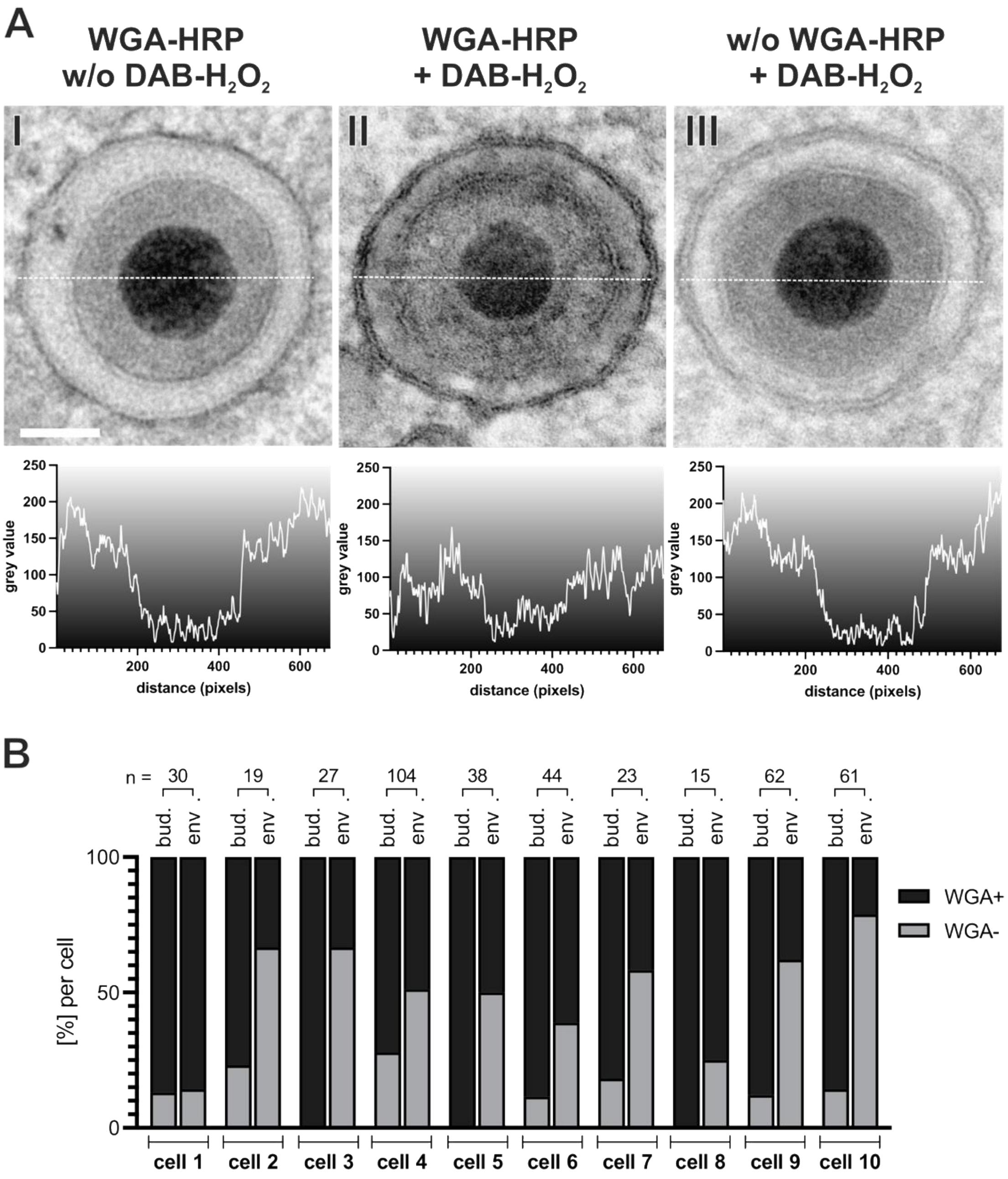

Figure S5: Quantification of secondary envelopment at WGA-positive and WGA-negative membranes. (A) Enveloped capsids after treatment with WGA-HRP but without DAB-H_2O_2 (I), treated with WGA-HRP and DAB-H_2O_2 (II) and treated without WGA-HRP but with DAB-H_2O_2 (III). The grey value profiles measured along the dashed lines are shown below: WGA-negative virions exhibit a brighter space between the envelope and the vesicle membrane compared to the tegument (I and III). WGA-positive virions exhibit a darker or similarly dark space (II). (B) Results from Table 1 visualized per cell. Categorization of WGA-positive (WGA+) and WGA-negative (WGA-) capsids in 10 fibroblasts, dependent on their envelopment stage budding (bud.) or enveloped (env.). The total number of budding and enveloped capsids per cell, respectively, were set to 100%. n= total number of capsids/cell. “Cell 7” corresponds to the cell displayed in Figure 6.

Figure S6

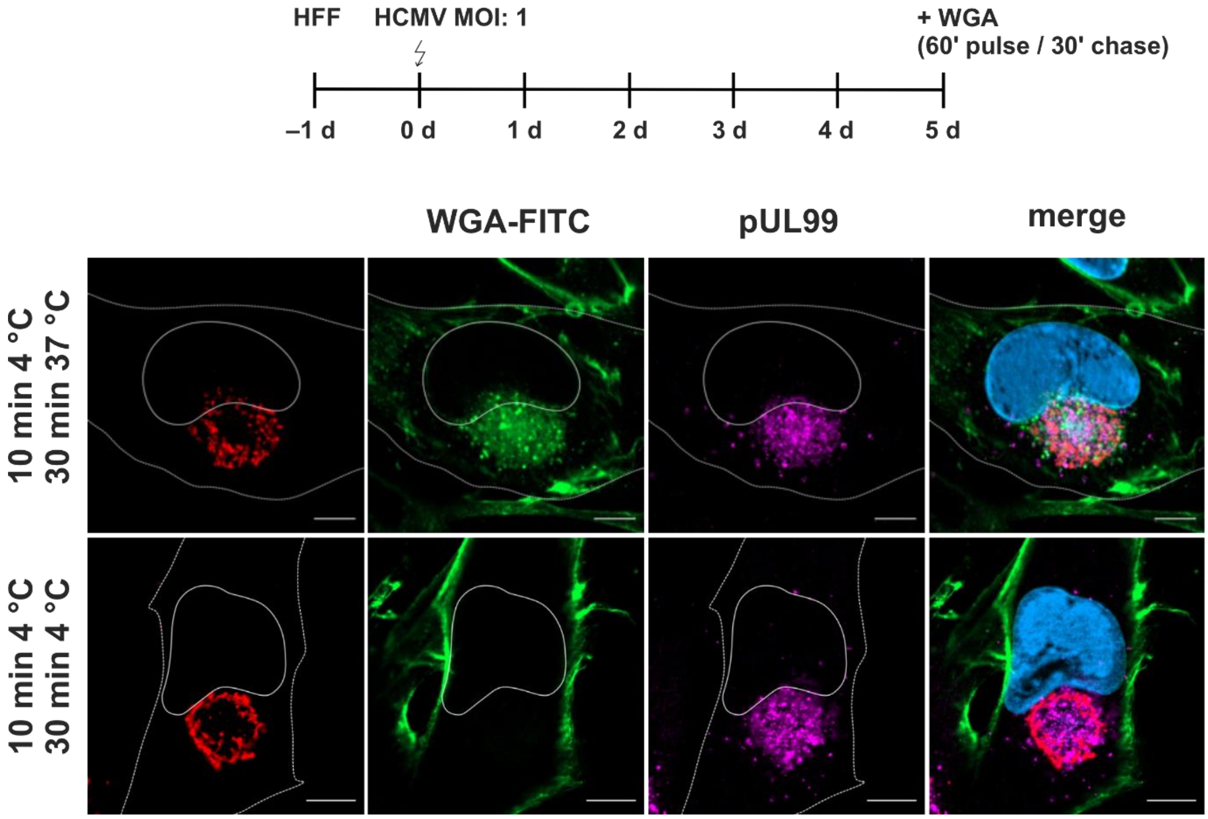

Figure S6: Internalization of WGA-FITC in HCMV-infected fibroblasts at 120 hpi after shorter labelling times. Infected fibroblasts were incubated with WGA-FITC (20 µg/ml) for 10 minutes by 4°C followed by a 30 minutes chase at either 37°C or 4°C. Performing the chase at 4°C inhibits the WGA-FITC internalization into the cell, leading to its accumulation at the plasma membrane (green). After reactivation of endocytosis by a temperature shift to 37°C, WGA-FITC accumulates at the cVAC, marked by the viral protein pUL99 (magenta) and delimited by the cis-Golgi protein marker GM130 (red). Cell nuclei were stained with DAPI (blue). Scale bars, 10 µm.
